# Engineered immune-driven theranostics for clinical cardiology

**DOI:** 10.1186/s40779-025-00664-6

**Published:** 2025-11-05

**Authors:** Jing-Ben Zheng, Xiao-Ye Li, Ji-Min Zhu, Cheng Liu, Xiao-Tian Song, Bin Wang, Yue Deng, Yu-Xiao Feng, Qi Wang, Juan Liu, Heng Dong, Xing-Jie Liang, Yuan Huang

**Affiliations:** 1 Nanjing Stomatological Hospital, Affiliated Hospital of Medical School, Research Institute of Stomatology, Nanjing University, Nanjing, 210008 China; 2 Department of Gastroenterology and Hepatology and Shanghai Institute of Liver Diseases, Zhongshan Hospital, Fudan University, Shanghai, 200032 China; 3 Department of Obstetrics and Gynecology, Renmin Hospital of Wuhan University, Wuhan, 430060 China; 4 Department of Immunology, Hebei Medical University, Shijiazhuang, 050017 China; 5 Department of Immunology, Binzhou Medical University, Yantai, 264100 Shandong China; 6 Hypertension Center, Fuwai Hospital, State Key Laboratory of Cardiovascular Disease, National Center for Cardiovascular Diseases, Chinese Academy of Medical Sciences and Peking Union Medical College, Beijing, 100730 China; 7 Department of Chemical Engineering, Tsinghua University, Beijing, 100084 China; 8 Hepato-Pancreato-Biliary Center, Beijing Tsinghua Changgung Hospital, & Key Laboratory of Digital Intelligence Hepatology, Ministry of Education, School of Clinical Medicine, Tsinghua Medicine, Tsinghua University, Beijing, 102218 China; 9 CAS Key Laboratory for Biomedical Effects of Nanomaterials and Nanosafety, CAS Center for Excellence in Nanoscience, National Center for Nanoscience and Technology of China, Beijing, 100190 China; 10 State Key Laboratory of New Pharmaceutical Preparations and Excipients, Hebei University, Baoding, 071000 Hebei China; 11 School of Biomedical Engineering, Guangzhou Medical University, Guangzhou, 511436 China; 12 Department of Structural Heart Disease, National Center for Cardiovascular Disease, China/Fuwai Hospital, Chinese Academy of Medical Sciences/Peking Union Medical College, Beijing, 100037 China

**Keywords:** Nanotheranostics, Cardiovascular diseases, Immune therapy, Medical imaging, Inflammatory regulation

## Abstract

Immunotherapy for cardiovascular diseases (CVDs) holds great promise for precision management by modulating localized immune-inflammatory responses. The interplay between focal cardiovascular pathology and panvascular disease, necessitates highly integrated therapeutic strategies. Nano-technology-based theranostic platforms address this challenge by enabling both regulation and real-time imaging of immune cell activity within cardiovascular lesions. These functional nanotherapy systems not only halt disease progression at pathological sites but also reduce secondary cardiovascular events driven by shared inflammatory mechanisms. Additionally, nanoplatform-based dynamic visualization of immune cell responses facilitates adaptive, personalized interventions. This review introduces the role of immune cells in CVDs. It summarizes recent advances in nanomaterial-based immunomodulation strategies, including mechanisms of immune regulation, enhanced imaging, and therapeutic applications in atherosclerosis, myocardial infarction, ischemic stroke, abdominal aortic aneurysm, and myocarditis. Collectively, this integrated nanotheranostic paradigm establishes a robust foundation for the next generation of cardiovascular precision medicine.

## Background

Cardiovascular diseases (CVDs) encompass a broad spectrum of disorders involving structural and functional defects of the heart and vasculature, including arteries, veins, and capillaries [1, 2]. CVDs compromise blood supply to vital organs such as the heart, brain, kidneys, and limbs, and remain a leading cause of morbidity and mortality worldwide [3–5]. Within this spectrum, atherosclerotic cardiovascular diseases (ASCVDs), including atherosclerosis (AS), myocardial infarction (MI), ischemic stroke (IS), and aneurysms, share AS as a common pathological substrate and constitute prototypical CVD entities, while non-atherosclerotic conditions such as myocarditis also account for a substantial proportion of cases [6–8]. Given the systemic, panvascular nature of CVDs, effective management often requires an integrated therapeutic strategy that targets AS as the core lesion while concurrently addressing the broader spectrum of cardiovascular disorders [9–11].

Extensive evidence identifies inflammation as a critical driver of CVD progression, including MI, hypertension, AS, IS, diabetic cardiomyopathy, and heart failure [12–16]. Vascular injury, such as endothelial dysfunction and ischemia-reperfusion injury (IRI), disrupts tissue homeostasis and triggers the release of pro-inflammatory cytokines, including interferon (IFN) -γ, interleukin (IL)-1β, IL-6, and tumor necrosis factor (TNF)-α. These mediators act through multiple signaling pathways, such as adenosine 5’-monophosphate-activated protein kinase, Janus-activated kinase (JAK)-signal transducer and activator of transcription 3 (STAT3), nuclear factor kappa-B (NF-κB), and phosphatidylinositol 3-kinase (PI3K)/protein kinase B (Akt) [17–22]. Concurrently, Toll-like receptor (TLR) upregulation on endothelial cells (ECs) enhances chemokine expression, driving the recruitment of immune cells and localized tissue damage [23, 24]. Thus, inflammation not only initiates vascular injury but also establishes a molecular environment that directly governs immune cell activation and recruitment, highlighting the close interplay between inflammatory mediators and immune responses in CVDs [25, 26]. Cytokine signaling further stimulates fibroblasts, smooth muscle cells (SMCs), and ECs, perpetuating a pro-inflammatory milieu [27, 28]. After vascular injury, endothelial activation and platelet complement signaling establish chemokine gradients and upregulate adhesion molecules, thereby recruiting immune cells to the lesion [29–31]. In the acute phase, these immune cells clear debris and coordinate repair; when activation is excessive or prolonged, they propagate thrombo-inflammation, extracellular matrix (ECM) proteolysis, and adverse remodeling that accelerates CVDs progression [32]. Immune cells, including macrophages, neutrophils, T cells, and B cells, regulate cytokine and protease release, phagocytose debris, and undergo phenotypic switching or programmed death, thereby orchestrating vascular remodeling and ECM turnover [33–36]. Their interactions with ECs and fibroblasts dynamically influence fibrogenesis, cellular proliferation, and vascular repair, underscoring the pivotal role of immune modulation throughout CVD initiation, progression, and resolution [37, 38].

Despite significant mechanistic insights, translating immune regulation into effective therapies remains a formidable challenge. Advances in nanotechnology provide promising solutions by enabling targeted delivery and precise imaging of immune and vascular processes. Nanomaterials ranging from 6 to 100 nm can evade rapid renal clearance [39], prolong systemic circulation, and achieve favorable biodistribution with controllable pharmacokinetics [40]. Their adjustable physicochemical properties allow for tailored composition and surface modification, facilitating targeted delivery, penetration across biological barriers, and reduced immune clearance [41, 42]. These features have already shown substantial clinical benefits in cancer immunotherapy [43]. Nanoplatforms hold comparable promise in CVDs, owing to shared mechanisms of immune modulation.

**Fig. 1:**
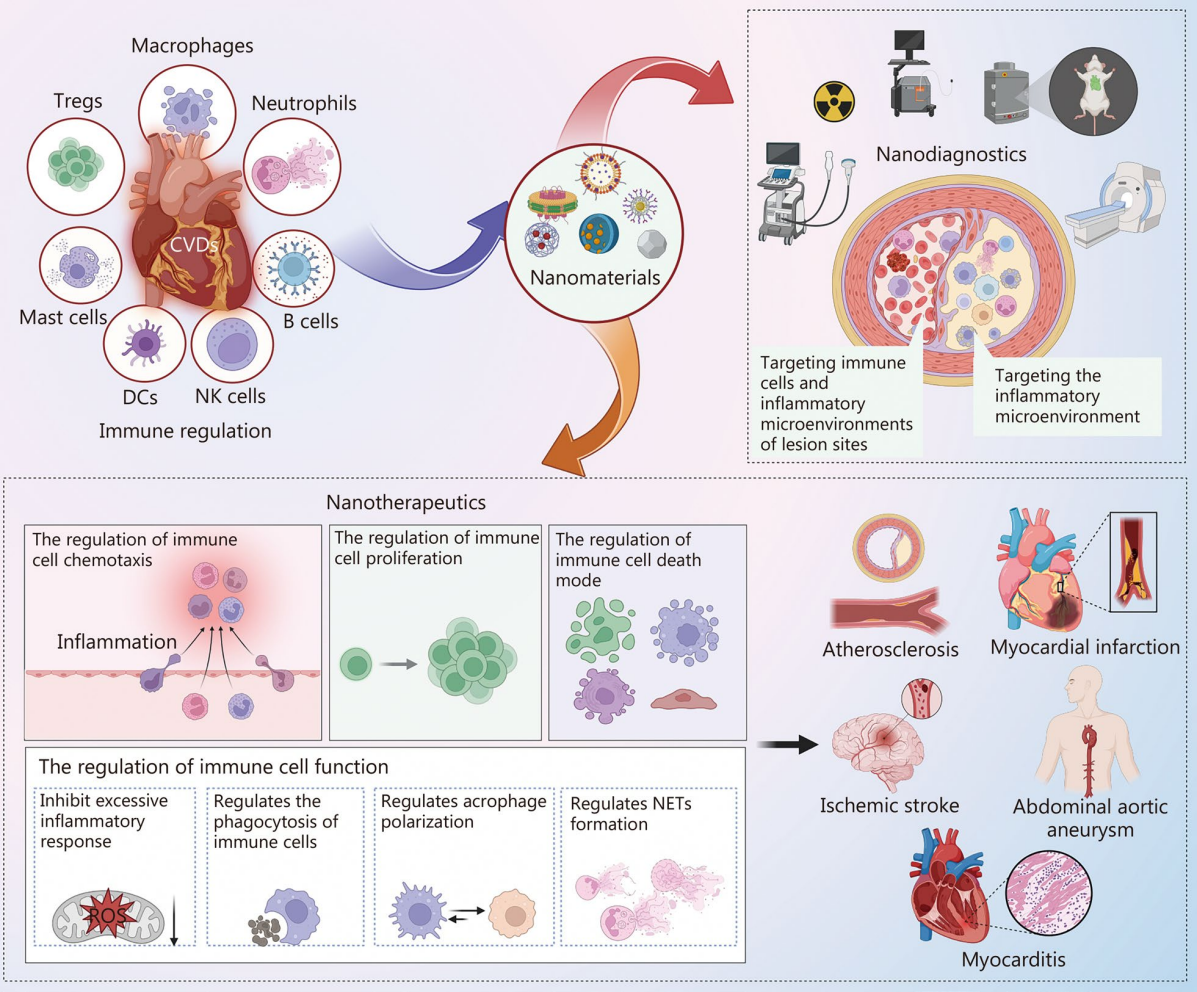
Schematic overview of engineered immune-driven nanotheranostics for clinical cardiology. Immune cells, including macrophages, neutrophils, regulatory T cells (Tregs), mast cells, dendritic cells (DCs), natural killer (NK) cells, and B cells play critical roles in cardiovascular diseases (CVDs). Nanomaterials modulate these immune responses to achieve both nanodiagnostic and nanotherapeutic outcomes. Nanodiagnostic platforms enable targeted imaging of immune cells and inflammatory microenvironments within cardiovascular lesions, supporting precise disease monitoring. Nanotherapeutics regulate multiple aspects of immune activity, including chemotaxis, proliferation, death modes, and functional responses such as excessive inflammation, phagocytosis, macrophage polarization, and neutrophil extracellular trap (NETs) formation. These integrated strategies offer potential applications in major cardiovascular pathologies, including atherosclerosis, myocardial infarction, ischemic stroke, abdominal aortic aneurysm, and myocarditis, thereby establishing a foundation for precision cardiovascular medicine

Nanomaterials can regulate cytokine signaling, gene expression, immune cell recruitment, and oxidative stress to mitigate vascular inflammation and promote repair, while also enhancing immune cell and inflammatory microenvironment targeted cardiovascular imaging for accurate diagnosis. This review first outlines the roles of immune cells in CVDs, then discusses the mechanisms by which nanomaterials modulate immune responses, including their effects on cell recruitment, proliferation, differentiation, and secretory activity. We further explore the applications of nanomaterials across major CVD subtypes, such as AS, MI, IS, aneurysms, and myocarditis, and examine their contributions to diagnosis, treatment, and prevention (Fig. 1). Finally, we address the challenges of clinical translation and highlight future directions for nanotheranostic strategies in precision cardiovascular medicine.

## The role of immune cells in CVDs

Immune cells are central orchestrators of the initiation, progression, and resolution of CVDs. Their multifaceted interactions with vascular, stromal, and parenchymal cells shape the inflammatory and reparative microenvironment that determines disease outcome. A comprehensive understanding of the mechanisms by which distinct immune cell subsets contribute to the pathogenesis of CVDs provides the foundation for engineering targeted nanodiagnostic and nanotherapeutic interventions.

### Macrophages in CVDs

**Fig. 2:**
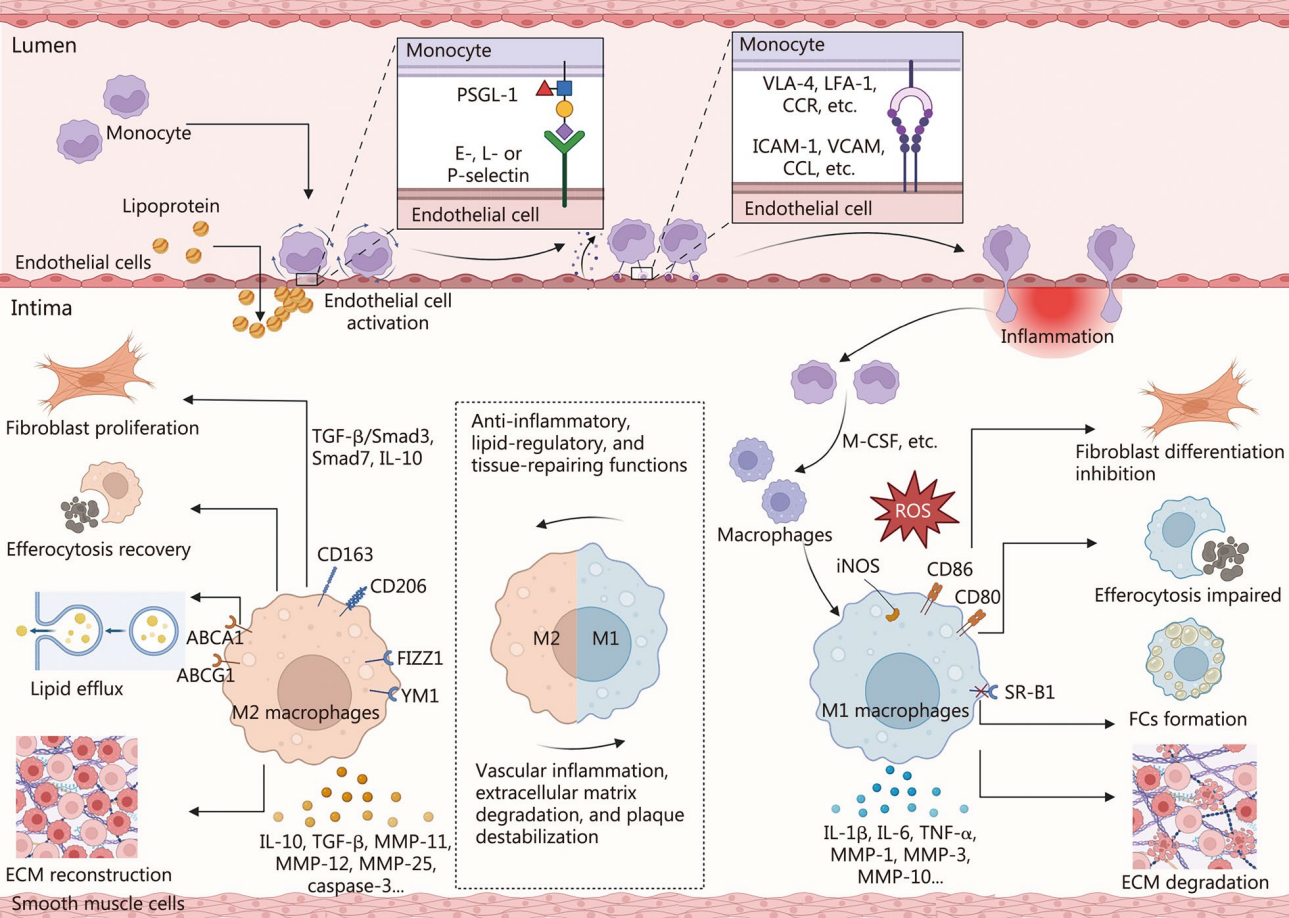
Role of macrophages in CVDs. Monocytes adhere to and transmigrate across activated ECs via selectin- and integrin-mediated interactions. Under inflammatory stimuli, they differentiate into pro-inflammatory M1 macrophages, which drive vascular inflammation, FC formation, impaired efferocytosis, ECM degradation, and plaque destabilization through ROS and cytokine release. As inflammation resolves, macrophages polarize toward the M2 phenotype, exerting anti-inflammatory, lipid-regulatory, and tissue-repairing functions by enhancing cholesterol efflux, stimulating fibroblast proliferation, and promoting ECM reconstruction. PSGL-1 P-selectin glycoprotein ligand-1, CCR C-C motif chemokine receptor, CCL C-C motif chemokine ligand, LFA-1 lymphocyte function-associated antigen-1, VLA-4 very late antigen-4, VCAM-1 vascular cell adhesion molecule-1, ICAM-1 intercellular adhesion molecule-1, M-CSF macrophage colony-stimulating factor, DC dendritic cell, M1 macrophage classically activated macrophage phenotype, M2 macrophage alternatively activated macrophage phenotype, iNOS inducible nitric oxide synthase, IL Interleukin, TNF-α tumor necrosis factor-α, NLRP3 NOD-like receptor family pyrin domain containing 3, SMC smooth muscle cell, MMP matrix metalloproteinase, ABCA1 ATP-binding cassette subfamily A1, ABCG1 ATP-binding cassette subfamily G1, SR-B1 scavenger receptor class B type 1, Arg-1 arginase-1, FIZZ1 found in inflammatory zone 1, YM1 chitinase 3-like 1, TGF-β transforming growth factor-β, CVDs cardiovascular diseases, ECs endothelial cells, M1 macrophages classically activated macrophage phenotype, FCs foam cells, ECM extracellular matrix, ROS reactive oxygen species, M2 macrophages alternatively activated macrophage phenotype

Macrophages, derived from circulating monocytes, regulate CVDs through lipid metabolism, inflammatory signaling, efferocytosis, oxidative stress, and fibrotic processes (Fig. 2). Upon vascular injury, monocytes tether to the endothelium by engaging P-selectin glycoprotein ligand-1 (PSGL-1) with E-, L-, or P-selectins expressed on ECs. Subsequent activation of C-C motif chemokine receptor (CCR) 2 by EC-derived C-C motif chemokine ligand (CCL) 2 induces conformational activation of integrins, such as lymphocyte function-associated antigen-1 (LFA-1) and very late antigen-4 (VLA-4), facilitating firm adhesion to vascular cell adhesion molecule (VCAM)-1 and intercellular cell adhesion molecule (ICAM)-1. Monocytes then transmigrate across the vascular wall and differentiate into macrophages, thereby amplifying local inflammation [44–50]. In early AS, macrophage colony-stimulating factor (M-CSF) and other differentiation signals drive monocyte maturation into macrophage- and dendritic cell (DC)-like phenotypes [51, 52].

Macrophages exhibit remarkable plasticity and can polarize into classically activated macrophage phenotype (M1) or alternatively activated macrophage phenotype (M2) subtypes. M1 macrophages express surface markers such as CD80, CD86, and inducible nitric oxide synthase (iNOS), and are predominantly associated with pro-inflammatory responses [34, 53, 54]. They release cytokines, including IL-1β, IL-6, and TNF-α, amplifying inflammation via the NOD-like receptor thermal protein domain-associated protein 3 (NLRP3) inflammasome/IL-1β axis. These mediators promote SMCs proliferation, ECs activation, and vascular permeability, which in turn recruit additional monocytes to lesion sites and reinforce inflammatory loops [55–58]. Moreover, M1 macrophages secrete matrix metalloproteinases (MMPs) such as MMP-1, MMP-3, and MMP-10, driving ECM degradation [59]. Endothelial dysfunction further contributes to this phenotype by increasing local reactive oxygen species (ROS) generation, reducing nitric oxide (NO) bioavailability, and activating redox-sensitive pathways, including NF-κB and noncanonical Wnt signaling, thereby favoring M1 polarization and matrix destabilization [60, 61]. A hallmark of macrophage dysfunction in AS is their transformation into foam cells (FCs) through uptake of apolipoprotein B-containing lipoproteins (apoB-LPs) [62–65]. Impaired cholesterol efflux, due to deficiencies in ATP-binding cassette transporters ATP-binding cassette protein A1 (ABCA1) and ATP-binding cassette protein G1 (ABCG1), as well as scavenger receptor class B type 1 (SR-B1), exacerbates lipid accumulation and FC formation [66–68]. These lipid metabolic disturbances activate endoplasmic reticulum stress and apoptotic pathways, leading to MMPs release, ECM breakdown, and macrophage apoptosis [69]. Under normal conditions, macrophages clear apoptotic cells via efferocytosis, a process mediated by SR-B1 through Src/PI3K/Ras-related C3 botulinum toxin substrate 1 (Rac1) signaling, thereby limiting necrotic core expansion and resolving inflammation [70, 71]. However, dysregulated lipid metabolism impairs efferocytosis through competitive inhibition of recognition receptors by lipids, suppression of bridging molecules, upregulation of “don’t eat me” signals, and mitochondrial dysfunction, collectively accelerating lesion progression [34].

In contrast, M2 macrophages display markers including CD163, CD206, arginase-1 (Arg-1), found in inflammatory zone 1 (FIZZ1), and chitinase 3-like 1 (YM1). They exert reparative functions through secretion of anti-inflammatory cytokines such as IL-10 and transforming growth factor-β (TGF-β) [72, 73]. These mediators attenuate pro-inflammatory cascades, reduce MMP-9 and MMP-2 activity, and increase MMP-11, MMP-12, and MMP-25 expression, thereby balancing ECM turnover [59, 74]. M2 macrophages also promote cholesterol efflux by upregulating ABCA1 and ABCG1 and induce apoptosis of inflammatory cells via caspase-3 activation [75–77]. Beyond immune regulation, M2 macrophages directly influence fibroblasts via the TGF-β/mothers against decapentaplegic homolog 3 (Smad3) axis, stimulating fibroblast migration, transdifferentiation into myofibroblasts, and synthesis of collagen and fibronectin. Smad7-mediated feedback further supports fibroblast proliferation and fibrogenesis during tissue repair [78, 79]. Importantly, IL-10 facilitates fibrotic remodeling by promoting fibroblast-to-myofibroblast transition, cardiac macrophage polarization, and osteopontin (OPN) expression [80, 81].

Macrophages exert a dual role in CVDs by shaping immune responses through phenotype switching and the corresponding changes in cytokine secretion and cellular functions. On the one hand, pro-inflammatory M1 macrophages, together with FCs derived from dysregulated lipid metabolism, drive vascular inflammation, ECM degradation, and plaque destabilization, thereby accelerating disease progression. On the other hand, macrophage-mediated efferocytosis by M1 cells, as well as the anti-inflammatory, lipid-regulatory, and tissue-repairing functions of M2 macrophages, contribute to the resolution of inflammation and lesion stabilization. Importantly, macrophages exhibit both passive and active uptake of nanomaterials, making them ideal targets for therapeutic nanointerventions. Their multifunctional roles in CVD pathogenesis provide multiple points of entry for nanomedicine-based modulation, with the overarching therapeutic objective of limiting lesion destruction while promoting tissue repair [82, 83]. Based on these principles, several potential strategies for nanomaterial targeting have been proposed: 1) reprogramming the inflammatory microenvironment within lesions, 2) inhibiting EC-mediated monocyte recruitment to reduce macrophage infiltration, 3) regulating lipid metabolism to prevent FC formation, and 4) suppressing M1 polarization while enhancing M2 differentiation and function to control local inflammation, minimize tissue injury, and accelerate repair [84–86].

### Neutrophil in CVDs

**Fig. 3:**
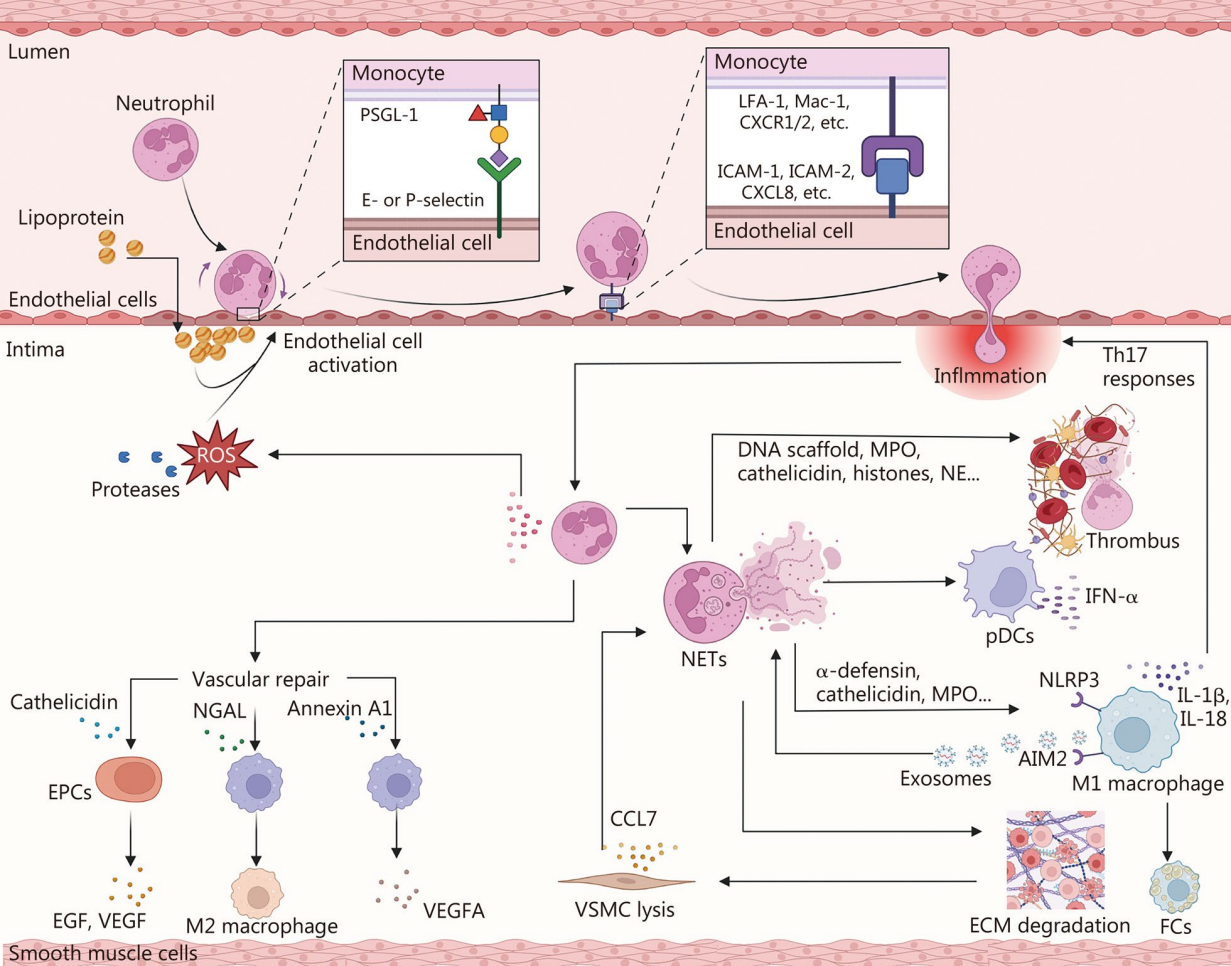
Role of neutrophils in CVDs. Neutrophils adhere to and transmigrate across activated endothelial cells via selectin- and integrin-mediated interactions. Activated neutrophils release ROS, proteases, and granule proteins, promoting endothelial dysfunction, FC formation, ECM degradation, and inflammation. Neutrophil extracellular traps (NETs) further amplify immune activation through inflammasomes and pDC-derived IFN-α, contributing to thrombus formation and Th17 responses. Conversely, neutrophil-derived mediators such as cathelicidin, NGAL, and annexin A1 promote vascular repair via EPC activation, M2 polarization, and angiogenesis. PSGL-1 P-selectin glycoprotein ligand-1, CXCL C-X-C motif chemokine ligand, CXCR C-X-C motif chemokine receptor, LFA-1 lymphocyte function-associated antigen-1, Mac-1 macrophage-1 antigen, ICAM intercellular adhesion molecule, LDL low-density lipoprotein, MPO myeloperoxidase, NETs neutrophil extracellular traps, NE neutrophil elastase, pDCs plasmacytoid dendritic cells, IFN-α interferon-α, NLRP3 NOD-like receptor family pyrin domain containing 3, AIM2 absent in melanoma 2, IL interleukin, VSMCs vascular smooth muscle cells, CCL C-C motif chemokine ligand, MMPs matrix metalloproteinases, FPR2 formyl peptide receptor 2, EGF epidermal growth factor, VEGF vascular endothelial growth factor, VEGFA vascular endothelial growth factor A, MerTK MER tyrosine kinase, CVDs cardiovascular diseases, ROS reactive oxygen species, FCs foam cells, ECM extracellular matrix, pDC plasmacytoid dendritic cell, IFN-α interferon-α, Th T helper, NGAL neutrophil gelatinase-associated lipocalin, EPC endothelial progenitor cell, M1 macrophage classically activated macrophage phenotype, M2 macrophage alternatively activated macrophage phenotype

Neutrophils are critical regulators of CVDs (Fig. 3). Their recruitment follows a well-orchestrated process of rolling, adhesion, and transmigration. Initially, neutrophils engage P-selectin glycoprotein ligand-1 (PSGL-1) with endothelial P- and E-selectins, initiating rolling. Subsequently, endothelial-derived C-X-C motif ligand (CXCL) 8 activates neutrophil C-X-C motif receptors (CXCR1/2), triggering integrin activation (LFA-1 and macrophage-1 antigen, Mac-1). This enables firm adhesion to ICAM-1 and -2, followed by transendothelial migration into the vascular wall, where neutrophils amplify inflammatory responses [87, 88]. Within atherosclerotic plaques, neutrophils secrete ROS and proteases that disrupt the endothelial barrier and degrade the ECM, thereby promoting immune cell infiltration and low-density lipoprotein (LDL) translocation from the lumen into the intima [89]. Granule proteins such as α-defensin, cathelicidin, and myeloperoxidase (MPO) further skew macrophages toward an M1 phenotype, accelerating FCs formation and amplifying local inflammation [90–92].

A hallmark of neutrophil-mediated pathology in CVDs is the formation of neutrophil extracellular traps (NETs). These web-like structures, composed of DNA, histones, neutrophil elastase (NE), MPO, serine proteases (cathepsin G, NE, and proteinase 3), IL-1α, and tissue factor, are potent inducers of vascular injury and thrombosis [93, 94]. NETs activate plasmacytoid DCs (pDCs) to secrete pro-atherogenic IFN-α, while stimulating macrophages through NLRP3 and melanoma 2 (AIM2) inflammasomes to produce IL-1β and IL-18 [95–97]. This cytokine milieu amplifies inflammation and establishes a feed-forward loop that sustains neutrophil activation and further NETs release [98–100]. IL-1β also drives Th17 responses, enhancing immune cell recruitment to atherosclerotic lesions [101]. Crosstalk between neutrophils and macrophages is reinforced through exosomal signaling, such as macrophage-derived exosomes enriched in miR-146a, which induce oxidative stress in neutrophils by suppressing superoxide dismutase (SOD) 2, thereby triggering NETs formation [102]. Moreover, vascular SMCs (VSMCs) contribute to this interplay, such as CCL7 secreted by activated VSMCs stimulates neutrophil activation, while NETs-associated histone H4 induces VSMC lysis, and neutrophil-derived MMPs exacerbate ECM degradation and VSMC death [89, 94, 103]. Beyond vascular inflammation, NETs play a central role in thrombosis by providing a structural scaffold enriched with tissue factor, coagulation factor XII, histones H3/H4, and fibrinogen that trap platelets and erythrocytes, reinforcing thrombus formation [104, 105].

Despite their pathogenic roles, neutrophils also contribute to vascular repair. During angiogenesis, neutrophil-derived cathelicidin deposited at injured arterial sites recruits endothelial progenitor cells (EPCs) via N-formyl peptide receptor 2 (FPR2) signaling, promoting re-endothelialization and paracrine release of growth factors such as epidermal growth factor (EGF) and vascular endothelial growth factor (VEGF) [106, 107]. Additionally, neutrophils secrete annexin A1, which binds FPR2 on macrophages to drive a pro-angiogenic phenotype and vascular endothelial growth factor A (VEGFA) production [108]. In fibrotic repair, neutrophil gelatinase-associated lipocalin (NGAL) stimulates macrophages through a MER tyrosine kinase (MerTK)-dependent mechanism, inducing a pro-repair phenotype that facilitates ECM remodeling [109]. These findings highlight the dual functionality of neutrophils in vascular regeneration and remodeling.

Overall, neutrophils serve as auxiliary yet versatile players in CVDs. Non-NETotic neutrophils disrupt vascular homeostasis by promoting monocyte infiltration and dysregulated lipid metabolism, thereby accelerating FCs formation. NETosis, however, acts as a powerful amplifier of vascular pathology by sustaining inflammation, inducing oxidative stress, mediating immune crosstalk, and driving thrombosis. Although neutrophils are classically viewed as terminally differentiated cells lacking plasticity, evidence suggests that subsets such as CXCR4^high^VEGFR^+^CD49d^+^ neutrophils exhibit tissue repair and pro-angiogenic functions [106, 108, 110]. This functional heterogeneity implies that neutrophil roles in CVDs are dynamic and disease-stage dependent.

Nanotherapeutic strategies targeting neutrophils aim to balance their destructive and reparative functions. Approaches include inhibition of neutrophil recruitment and adhesion, regulation of local oxidative stress, and suppression of NETosis. These strategies can be achieved using small interfering RNA (siRNA)-mediated chemokine silencing, CCR blockade, pro-inflammatory neutrophil apoptosis induction, ROS scavenging, and pharmacological inhibition of NET formation [111–113]. Collectively, such interventions provide a multi-pronged framework for nanomedicine-based modulation of neutrophils to limit tissue injury while promoting vascular repair.

### Regulatory T cells (Tregs)

**Fig. 4:**
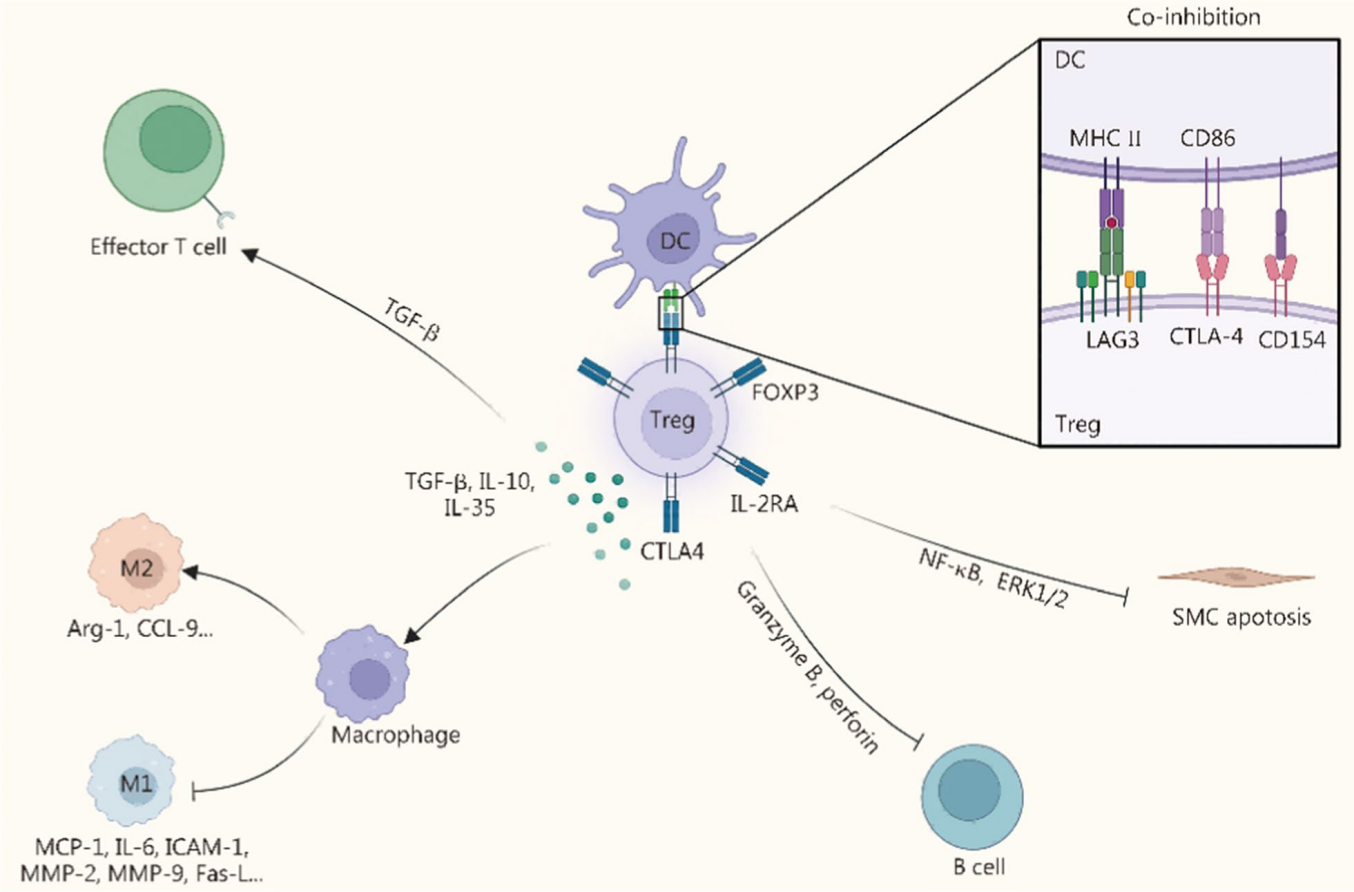
Regulatory roles of Tregs in cardiovascular diseases. Tregs exert immunosuppressive effects in CVDs through the secretion of anti-inflammatory cytokines (e.g., TGF-β, IL-35, IL-10) and the expression of inhibitory molecules such as CTLA-4. By suppressing APCs and effector T cells, Tregs attenuate local immune activation. They also modulate macrophage polarization by promoting M2 phenotypes while inhibiting M1-driven inflammation, thereby reducing pro-inflammatory cytokines and ECM degradation. In addition, Tregs limit B-cell-mediated antibody production, inhibit SMC apoptosis via NF-κB and ERK1/2 pathways, and collectively contribute to vascular protection and tissue homeostasis. Tregs regulatory T cells, CVDs cardiovascular diseases, TGF-β transforming growth factor-β, IL interleukin, CTLA-4 cytotoxic T-lymphocyte**-**associated protein 4, APCs antigen-presenting cells, M2 macrophage alternatively activated macrophage phenotype, M1 macrophage classically activated macrophage phenotype, ECM extracellular matrix, SMC smooth muscle cell, NF-κB, nuclear factor kappa-B, ERK1/2 extracellular signal-regulated kinase 1/2, ICAM-1 intercellular cell adhesion molecule-1, MMP matrix metalloproteinase, MCP-1 monocyte chemoattractant protein-1, Fas-L Fas ligand, FOXP3 forkhead box protein P3, MHC major histocompatibility complex, LAG3 lymphocyte-activation gene 3, Treg regulatory T cell

In CVDs, Tregs play a central role among T-cell subsets by coordinating and modulating the activity of other effector immune cells to control inflammatory responses (Fig. 4) [114]. Tregs are defined by the expression of the transcription factor forkhead box protein P3 (FOXP3), IL-2 receptor subunit-α (IL-2RA), and cytotoxic T-lymphocyte-associated protein 4 (CTLA-4) [115]. Functionally, Tregs exert potent immunosuppressive effects through the secretion of anti-inflammatory cytokines such as TGF-β, IL-35, and IL-10, while reducing pro-inflammatory mediators including TNF-α, IL-1β, and monocyte chemoattractant protein-1 (MCP-1) [116, 117]. They also suppress the production of MMPs, such as MMP-2 and MMP-9, thereby attenuating ECM degradation and limiting vascular injury [118, 119].

The immunoregulatory functions of Tregs are mediated by both direct and indirect mechanisms. Directly, TGF-β secreted by Tregs binds to its receptor on effector T cells, inhibiting CD8^+^ T cell activation [120, 121]. Indirectly, Tregs modulate antigen-presenting cells (APCs), particularly DCs. For example, CTLA-4 on Tregs depletes CD86 from DCs through transendocytosis, reducing costimulatory signaling, while Treg-derived CD154 suppresses DC activation [122–124]. Additionally, the interaction of lymphocyte-activation gene 3 (LAG3) on Tregs with major histocompatibility complex (MHC)-II molecules on DCs initiates inhibitory signaling through immunoreceptor tyrosine-based motifs, preventing DC maturation and dampening their ability to stimulate effector T cells [125]. Tregs also exert significant effects on the innate immune compartment. TGF-β and IL-10 produced by Tregs inhibit M1 macrophage polarization while promoting M2 differentiation, thereby shifting the local immune balance toward tissue repair. This is accompanied by suppression of pro-inflammatory mediators [ MCP-1, IL-6, ICAM-1, MMP-2, MMP-9, Fas ligand (Fas-L)] and upregulation of anti-inflammatory and reparative markers (Arg-1, CCL-9) [126]. In vascular tissues, Tregs prevent SMCs apoptosis by blocking NF-κB and extracellular regulated protein kinases 1/2 (ERK1/2) pathway activation [126].

Furthermore, Tregs directly regulate B cells by eliminating antigen-presenting B cells via granzyme B- and perforin-dependent cytotoxicity, thereby reducing pathogenic antibody production [127, 128]. Beyond immune modulation, Tregs contribute to vascular and cardiac repair. They promote neovascularization, regulate fibroblast activity, and attenuate post-MI fibrosis, ultimately reducing cardiac stiffness and improving functional recovery in cardiomyopathies [129, 130]. These findings underscore the multifaceted therapeutic potential of Tregs in the context of CVDs.

Nanomaterial-based modulation of Tregs typically occurs in concert with other immune pathways. On the one hand, nanomaterials directly targeting Tregs often rely on the assistance of APCs, particularly tolerogenic DCs, which subsequently promote immune tolerance through Treg expansion, thereby preventing and repairing tissue damage in CVDs [131, 132]. On the other hand, nanomaterials directed at other immune cells can indirectly influence Treg activity by reshaping the inflammatory microenvironment [116, 133–135]. Such secondary regulation forms a positive feedback loop that enhances immune homeostasis and accelerates lesion recovery [136]. Accordingly, future nanomaterial strategies may advance along 2 axes: direct modulation of Tregs to prevent further amplification of inflammatory responses and mitigate local tissue damage; and regulation of upstream APCs to block subsequent Treg activation, thereby preventing additional tissue injury and fostering a local microenvironment conducive to tissue repair.

### Other immune cells in CVDs

Although macrophages, neutrophils, and T cells have been extensively studied in CVDs, other immune cell populations, including mast cells (MCs), DCs, natural killer (NK) cells, and B cells, also play crucial roles in shaping disease onset, progression, and resolution. While their diagnostic and therapeutic targeting in nanomedicine remains relatively underexplored, these cell types represent promising avenues for future investigation.

#### Mast cells (MCs) in CVDs

MCs, central regulators of innate immunity, mediate allergic reactions and host defense via the release of bioactive mediators such as platelet-derived growth factor A (PDGFA), TNF-α, TGF-β, and histamine, all of which influence cardiac physiology [137]. In CVDs, MCs promote vascular injury and plaque progression through enzymatic activities (tryptase, chymase) and pro-inflammatory cytokines (IL-6, IFN-γ), which induce protease expression in surrounding cells, resulting in subendothelial damage and immune cell recruitment [138–140]. In addition, MCs-derived tryptase and chymase also impair lipid homeostasis by degrading high-density lipoprotein (HDL) and reducing macrophage cholesterol efflux, while simultaneously enhancing LDL uptake by macrophages, thereby accelerating FCs formation [141, 142]. Moreover, TNF-α stimulates MMPs activity, exacerbating atherosclerotic plaque instability and promoting apoptosis of ECs and SMCs [143–147]. Histamine released by coronary MCs disrupts EC barrier integrity, facilitating lipoprotein translocation into the vessel wall [148–150]. Interestingly, MCs also exhibit pro-angiogenic functions. Tryptase stimulates endothelial tube formation and proliferation, while chymase promotes vascular remodeling via angiotensin II (Ang II)-dependent pathways [151–153]. Thus, MCs exert dual, context-dependent roles by driving inflammatory pathology while simultaneously supporting compensatory vascular adaptation, which highlights their complex contributions to CVD progression.

#### DCs in CVDs

As professional APCs, DCs serve as gatekeepers of adaptive immunity [154]. Necrotic tissue-derived epitopes and damage-associated molecular patterns (DAMPs), along with signals such as Ang II, induce phenotypic alterations in DCs, directing their differentiation toward either immunogenic or tolerogenic states [155]. Immunogenic DCs secrete pro-inflammatory cytokines (IL-12, IL-13, IFN-γ) and activate CD4^+^/CD8^+^ T cells, thereby amplifying immune-mediated tissue damage [156, 157]. In contrast, tolerogenic DCs suppress immune activation by inducing T-cell anergy/apoptosis and expanding Tregs, thus establishing immune homeostasis [158, 159]. This phenotypic plasticity positions DCs as pivotal regulators in CVDs, balancing pro-inflammatory injury and immunosuppressive repair. Given their dual roles, DCs represent attractive targets for nanomaterials designed to induce tolerogenic phenotypes and restore cardiovascular immune balance.

#### NK cells in CVDs

NK cells, belonging to the innate lymphoid cell family, express diverse CCRs that facilitate trafficking from the bone marrow to inflamed or ischemic cardiovascular tissues [160–162]. Pro-inflammatory NK responses are driven by the T-bet/IFN-γ/IL-12 axis, which establishes reciprocal activation with monocytes, amplifying immune cell recruitment and inflammatory cytokine secretion (e.g., IFN-γ, perforin, granzyme B) [163]. Conversely, NK cells also exert protective and reparative roles. By shaping an anti-inflammatory chemokine milieu, NK-derived IFN-γ can suppress fibrotic immune cell populations such as eosinophils, thereby limiting maladaptive fibrosis [164]. Furthermore, NK cells promote vascular regeneration and post-ischemic healing through integrin-α4β7/VCAM-1 interactions with ECs, contributing to revascularization and collagen deposition [165]. This functional dichotomy emphasizes NK cells as dynamic regulators, capable of both exacerbating inflammation and promoting repair depending on disease stage.

#### B cells in CVDs

B cells contribute to CVD progression and resolution through antibody production, cytokine secretion, and immunomodulation [166]. In AS, B cells are divided into B1 and B2 subsets with opposing functions. B1-derived immunoglobulin M (IgM) antibodies targeting oxidation-specific epitopes (OSEs) of oxidized LDL (ox-LDL) confer atheroprotection, whereas B2-derived IgG and IgE antibodies enhance atherogenesis [167, 168]. Beyond humoral responses, B cells interact with T cells, macrophages, and DCs, modulating immune crosstalk during cardiac repair [169]. Their cytokine secretion profile is context-dependent. IL-6 and TNF amplify inflammation, whereas IL-10 promotes resolution and repair [170, 171]. In MI, B cell-derived CCL-7 facilitates monocyte recruitment, exacerbating cardiac injury [172]. Thus, B cells also display dual roles, balancing pathogenic antibody-mediated responses and homeostatic regulation of tissue repair, underscoring their importance as modulators of cardiovascular immunity.

## Mechanisms of immune cell modulation by nanomaterials in CVDs

Nanomaterials exhibit remarkable potential in resolving localized inflammatory responses in CVDs, primarily by improving drug pharmacokinetics and enabling precise immune modulation. Upon systemic or local administration, nanomaterials preferentially accumulate at inflammatory sites either through passive targeting, such as enhanced permeability and retention (EPR) effects mediated by leaky vasculature, or through active targeting strategies that exploit ligand-receptor interactions on immune or ECs. These mechanisms significantly enhance subendothelial retention and therapeutic efficiency [173]. Targeting specificity is critical, as non-targeted formulations generally demonstrate markedly lower efficacy compared with engineered, targeted nanomaterials [174].

**Fig. 5:**
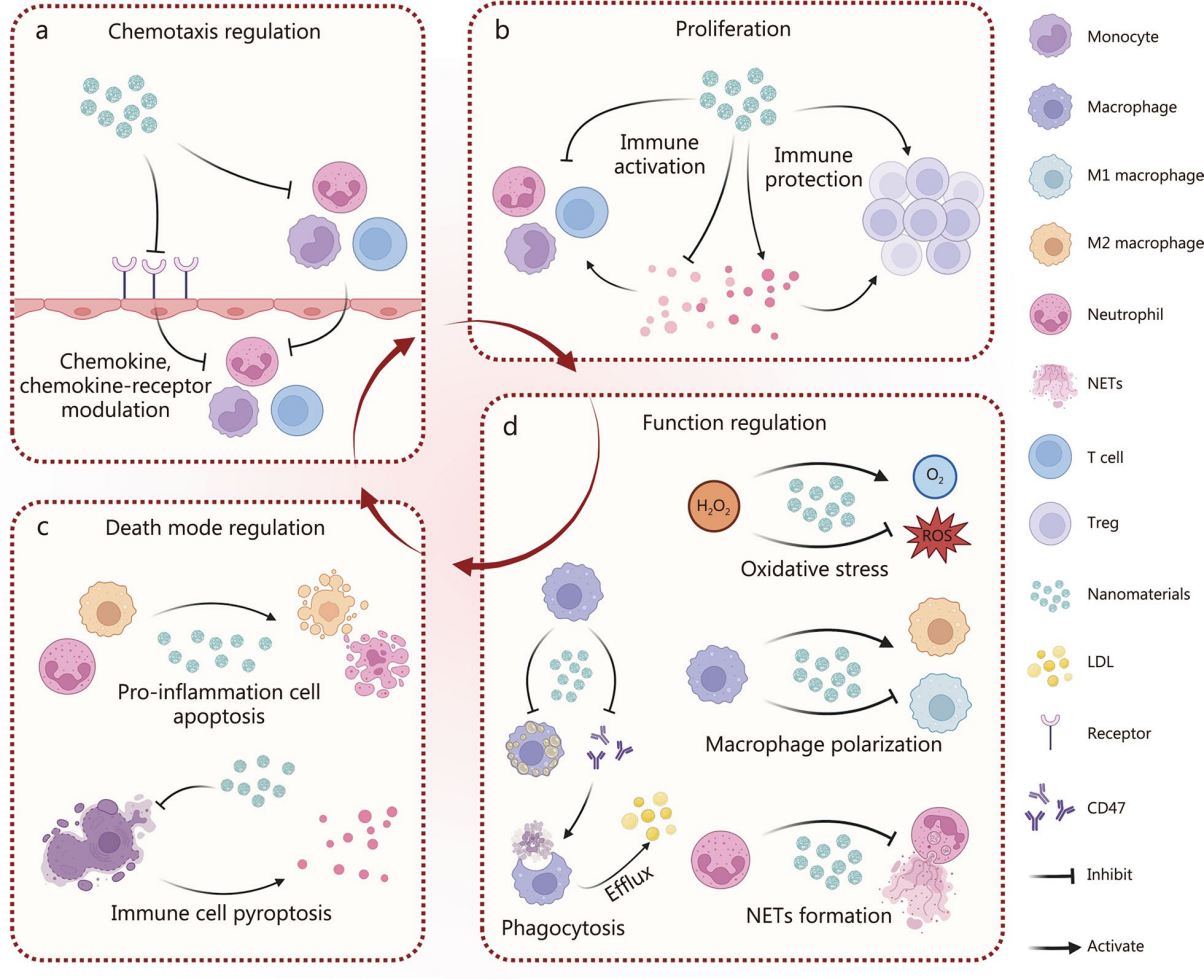
Comprehensive modulation of immune cell-mediated inflammatory responses by nanomaterials. **a** Nanomaterials can modulate chemokines and their receptors to effectively suppress the migration and infiltration of immunocytes, thereby attenuating inflammatory responses. Current strategies encompass gene silencing of adhesion molecules and chemokines, inhibition of associated signaling pathways, direct neutralization of chemokines, as well as the use of “cell hitchhiking” approaches to achieve precise targeted delivery. **b** Nanomaterials regulate immune cell proliferation, suppressing pro-inflammatory responses while promoting protective immune functions to modulate the progression of CVDs. **c** Nanomaterials regulate immune cell functions through multiple mechanisms, including inhibition of inflammatory signaling pathways, ROS scavenging, modulation of phagocytosis, promotion of M2 macrophage polarization, and suppression of NETs formation. These strategies collectively attenuate inflammation, facilitate tissue repair, and preserve cardiovascular function within interconnected immune regulatory networks. **d** Nanomaterials modulate immune cell death modes to alleviate inflammation and protect cardiovascular function. They can induce apoptosis while suppressing NETosis to reduce tissue injury and promote M2 macrophage polarization, and they can also inhibit pyroptosis to block inflammatory cascades and improve the local microenvironment. LDL low-density lipoprotein, Treg regulatory T cell, CVDs cardiovascular diseases, ROS reactive oxygen species, NETs neutrophil extracellular traps, M2 alternatively activated macrophage phenotype

The immunomodulatory potential of nanomaterials is largely determined by their physicochemical properties. By fine-tuning parameters such as size, shape, surface charge, chemical composition, and functional surface modifications, their biodistribution, circulation half-life, clearance rate, and cellular uptake can be optimized to maximize therapeutic benefit [175, 176]. For example, positively charged nanomaterials enhance interactions with negatively charged cell membranes, while ligand-functionalized surfaces enable selective delivery to macrophages, neutrophils, or T cells within cardiovascular lesions. Functionally, nanomaterials act as versatile platforms for delivering immunoregulatory agents, enabling precise control over immune cell behavior (Table 1) [177–202]. On the one hand, they directly modulate immune cell dynamics by regulating recruitment, proliferation, polarization, apoptosis, and cytokine secretion, thereby influencing the pathophysiological progression of CVDs. On the other hand, nanomaterials reshape the inflammatory microenvironment through spatiotemporally controlled drug release, which attenuates pathological inflammation while preserving reparative immune responses. This dual-action mechanism, by concurrent regulation of immune cell activity and restoration of microenvironmental homeostasis, underscores the multifaceted therapeutic promise of nanomaterials in CVDs. By integrating targeted delivery with immune modulation, nanomedicine offers a powerful and precise strategy for correcting immune dysregulation, stabilizing lesions, and promoting tissue repair in cardiovascular pathology (Fig. 5).

**Table 1:** Representative nanomaterials for nanotherapeutics in CVDs

Nanomaterials	Immune cell modulation	Class of nanomaterials	Targeted cells	CVDs	Targeting methods	Theragnostic mechanisms	Reference
siCAM^5^	Regulation of immune cell chemotaxis	Polymers	ECs	MI	Targeted gene	Silencing of ICAM-1, ICAM-2, VCAM-1, E-Selectin, and P-Selectin genes in quiescent ECs blocks neutrophil and leukocyte recruitment and reduces the activity of matrix-degrading plaque proteases	[177]
Nitavastatin-NPs		Polymers	Macrophages	AS	PLGA delivery	Inhibiting the activation of the NF-κB signaling pathway in monocyte-macrophages reduces the expression of MCP-1 and MMPs within the macrophages, thereby suppressing monocyte migration mediated by MCP-1/CCR2 signaling	[178]
VIN		Polymers	Neutrophils, macrophages	IS	The membrane of CXCR4-overexpressing mesenchymal stem cells	Integrating multiple functions such as blocking inflammatory chemotaxis, filtering peripheral inflammatory cells, promoting M2 polarization of activated brain-resident microglia, and scavenging ROS	[179]
^64^Cu-vMIP-II-Comb		Polymers	Macrophage	AS	vMIP-II	Targeting eight abnormally upregulated CCRs on macrophages in AS revealed plaque progression, including plaque size and the extent of macrophage infiltration, both of which correspond to elevated CCR concentrations	[180]
Leukosomes	Regulation of immune cell proliferation	Biomimetic nanomaterials	Macrophage	AS	MM	Targeting macrophages in AS for rapamycin delivery reduces the drug's undesirable systemic effects. This approach gains the advantages of immune evasion, long circulation, and targeted delivery, while also exerting therapeutic anti-inflammatory and anti-proliferative effects on the vascular wall, thereby effectively suppressing AS progression	[181]
P210-PAMs		Polymers	DCs	AS	P210’s proteoglycan-binding characteristics	The uptake of P210-PAM by DCs and its co-presentation with MHC-I molecules inhibited P210-specific CD4^+^ T cell proliferation and CD8^+^ T cell cytotoxic responses, while also promoting M2 polarization of macrophages	[182]
Exo-srIĸB	Inhibit excessive inflammatory response	Biomimetic nanomaterials	Neutrophils, macrophages	IRI	Exosomes derived from the human embryonic kidney 293 T cell line	Exo-srIκB inhibits NF-κB signaling activation by blocking the nuclear translocation of the NF-κB subunit p65. This thereby prevents the further escalation and amplification of the inflammatory response	[183]
VP-Gd-NF-NG		Polymers	ECs	AS	VP	After targeting ECs, siNF-κB reduces p65 expression, thereby enabling endothelial-targeted MRI imaging and visual anti-inflammatory therapy for AS	[184]
MM/ANPs		Biomimetic nanomaterials	Macrophage	IRI	MMs coated with the DSPE-PEG-RVG29 peptide	Effectively crosses the BBB and selectively accumulates in ischemic and inflamed regions. Enhancing SIRT3 activity inhibits NLRP3 inflammasome assembly, thereby suppressing inflammatory responses and pyroptosis	[185]
NE-ABR-Cu_x_O		Polymers	Neutrophils	MI	S100A8/A9 inhibitor ABR molecule	Cu_x_O NPs effectively scavenged various types of ROS, while ABR blocked the activation of excessive neutrophils infiltrating the infarct area. This dual approach synergistically inhibited the inflammatory immune response	[186]
BPNSs@PEG-S2P/R		Inorganic nanomaterials	Macrophage	AS	S2P peptide	After targeted accumulation in AS plaques, it inhibits macrophage burden, inflammatory responses, and oxidative stress within the plaque, thereby significantly reducing plaque area	[187]
MM NPs		Biomimetic nanomaterials	Neutrophils, macrophages	Acute lung injury	MM	Targeting miRNAs suppresses the inflammatory response of pulmonary macrophages and blocks macrophage-exacerbated NETosis, thereby inhibiting the release of local inflammatory molecules and the amplification of the inflammatory response	[188]
L-Ag/R		LNPs	DCs	MI	DOTAP	The NPs co-delivered antigens and rapamycin to DCs, inducing immune suppression. This triggered antigen-specific immune tolerance by generating tDCs and Tregs, and modulating macrophage polarization	[189]
Fer-aCD47@PHMSN	Regulates the phagocytosis of immune cells	Inorganic nanomaterials	CMs, macrophages	MI	Platelet membrane coating	aCD47 effectively suppresses ferroptosis in CMs. Simultaneously, it enhances macrophage-mediated phagocytosis of dead CMs, thereby inhibiting post-MI inflammation	[190]
CAR-M/β-CD LNP		Biomimetic nanomaterials	Macrophages	AS	CAR-M	The combination of CAR and NP engineering activated macrophage lipid efflux pumps and enhanced the phagocytic capacity of macrophages toward apoptotic cells that overexpress CD47 due to chronic inflammation	[191]
miR33-chNPs		Polymers	Macrophages	AS	chNPs	By greatly enhancing ABCA1 expression and cholesterol efflux in naive macrophages, it effectively targets AS plaque lesions	[192]
1 cM + GW3965		Polymers	Macrophages	AS	1 cM	The dual-pronged approach of targeting inflamed macrophages with a 1 cM large molecules, which leads to partial SR-A blockade, combined with GW3965 treatment, synergistically induced a significant depletion of ox-LDL within the macrophages	[193]
mDNP-LXR-L		Polymers	Macrophages	AS	mDNP	mDNP targets macrophages and adopts a dual approach, activating LXR while inhibiting SR-A. This action promotes the upregulation of ABCA1 and ABCG1, thereby reducing the formation of FCs and AS	[194]
Rb1/PDA nanogel	Regulates macrophage polarization	Polymers	Macrophages	MI	Benzyl borate bond	Leveraging ideal adhesive properties for the myocardial surface, it possesses multiple functions: scavenging ROS, protecting mitochondrial function, polarizing M2 macrophages, and promoting angiogenesis via a combination of internal and external spaces. This effectively mitigates MI and promotes recovery	[195]
MNPs/Alg hydrogels		Polymers	Macrophages	MI	N/A	By scavenging ROS, it significantly enhances CMs’ survival and promotes the polarization of macrophages to the M2 phenotype	[196]
M@A-F/E NCs		Polymers	Macrophages	IRI	MM	By crossing the BBB and acting on microglia, it exerts anti-inflammatory and antioxidant effects, promotes M2 polarization, and reduces the secretion of pro-inflammatory cytokines. This also confers anti-apoptotic properties on neuronal cells	[197]
LFPN		Polymers	Macrophages, ECs	MI	legumain	Regulation of macrophage polarization weakens the inflammatory response and promotes ECs’ angiogenesis	[198]
EVMS@R-HNC		Bionic nanomaterials	Macrophages, SMC	AAA	RGD sequence	The loaded EVMS, possessing an excellent ability to target macrophages and SMCs in AAA lesions, effectively inhibited M1-like macrophage polarization and preserved normal SMC contractile function	[199]
Col IV NPs	Regulates NETs formation	Polymers	Neutrophils	AS	Col IV	Leveraging the selective targeting of Col IV to the basement membrane of the lesion area, the NPs blocked local NETosis in AS, reduced NETs accumulation at the intimal lesion site, and improved endothelial continuity	[200]
PtIr nanozyme		Inorganic nanomaterials	Neutrophils	MI	N/A	PtIr nanozymes exhibit enhanced dual-enzyme activities, mimicking SOD and CAT, and protect CMs by scavenging ROS and suppressing NETs formation, thereby reducing acute inflammation and apoptosis	[201]
RC NPs	Regulation of immune cell death	Polymers	Neutrophils	MI	Hydrogen peroxide enzyme	In response to abnormally elevated H_2_O_2_ in the MI region, it rapidly releases roscovitine, which induces timely neutrophil apoptosis and inhibits neutrophil-mediated inflammation	[202]

*ECs* endothelial cells, *siCAM*^*5*^ siRNA-loaded nanoparticles targeting 5 endothelial adhesion molecules, endothelial cells, *MI* myocardial infarction, *ICAM* intercellular adhesion molecule, *VCAM* vascular cell adhesion molecule, *VIN* mesenchymal stem cell membrane-coated polydopamine core, *AS* atherosclerosis, *PLGA* poly(lactic-co-glycolic acid), *NF-κB* nuclear factor kappa-B, *MCP-1* monocyte chemoattractant protein-1, *MMP* matrix metalloproteinase, *CCR2* C-C chemokine receptor type 2, *IS* ischemic stroke, *CXCR* C-X-C chemokine receptor type, *M2* Alternatively activated macrophage phenotype, *ROS* reactive oxygen species, *vMIP-II* viral macrophage inflammatory protein-II, *MM* macrophage membrane, *leukosomes* MM derived protein integrated phospholipid vesicles, *DC* dendritic cell, *P210* apolipoprotein B-100 peptide 210, *P210-PAMs* self-assembling peptide amphiphile micelles loaded P210, *Exo-srIĸB* exosomal super-repressor inhibitor of NF-ĸB, *VP-Gd-NF-NG* a three-in-one nucleic acid nanogel, *IRI* ischemia-reperfusion injury, *VP* VCAM-1-targeting peptide, *NE* nanoemulsion, *BPNSs@PEG-S2P/R* resolvin D1-loaded S2P-peptide-PEGylated black phosphorus nanosheets, *L-Ag/R* liposomes containing MI-related antigens and rapamycin, *chNP* chitosan NPs, *BBB* blood-brain barrier, *DSPE* distearoyl phosphatidylethanolamine, *PEG* polyethylene glycol, *SIRT3* sirtuin 3, *NLRP3* NOD-like receptor family pyrin domain containing 3, *APNs* apelin-13, *ABR* S100A8/A9 inhibitor, *LNPs* lipid nanoparticles, *DOTAP* 1,2-dioleoyl-3-trimethylammonium-propane, *NPs* nanoparticles, *tDCs* tolerogenic dendritic cells, *PDA* polydopamine, *CMs* cardiomyocytes, *CAR-M* chimeric antigen receptor macrophage, *PHMSN* platelet membrane-coated hollow mesoporous silicon NPs, *Fer-aCD47@PHMSN* PHMSN loaded with Ferrostatin-1 and anti-CD47, *HPβ-CD* hydroxypropyl β-cyclodextrin, *LXR* liver X receptor, *ox-LDL* oxidized low-density lipoprotein, *mDNP* mannose-functionalized dendrimeric NP, *1 cM* 1 carboxylate amphiphilic macromolecules, *ABCA1* ATP-binding cassette subfamily A1, *ABCG1* ATP-binding cassette subfamily G1, *Rb1/PDA nanogel* nanogel ginsenoside Rb1-loaded polydopamine nanogel, *MNPs/Alg* melanin NPs/alginate, *M@A-F/E NCs* MM-cloaked phosphorus-dendrimer/fibronectin nanocomposites, *LFPN* legumain-guided ferulate-peptide nanofibers, *AAA* abdominal aortic aneurysm, *EVMS* everolimus, *EVMS@R-HNC* RGD-functionalized hepatitis B virus core nanocages encapsulating EVMS, *SMC* smooth muscle cell, *Col IV* collagen IV, *NETs* neutrophil extracellular traps, *PtIr nanozyme* bimetallic nanozyme composed of Pt and Ir, *N/A* not applicable, *RC-NPs* roscovitine- and catalase-loaded PLGA NPs, *SOD* superoxide dismutase, *CAT* catalase

### The regulation of immune cell chemotaxis

The migration and infiltration of immune cells represent pivotal events in the initiation and amplification of localized inflammatory cascades. During the early stages of inflammation, circulating monocytes, neutrophils, and T cells are recruited to sites of injury through chemotactic gradients shaped by inflammatory mediators and chemokine networks [203]. Once recruited, these cells undergo functional reprogramming [77, 204]. Monocytes differentiate into pro-inflammatory macrophage subsets, neutrophils initiate NETosis and release NETs, while T cells transition into activated effector phenotypes. Collectively, these processes establish a pro-inflammatory microenvironment that drives CVD progression [205].

One therapeutic strategy is to attenuate immune cell infiltration by targeting endothelial adhesion molecules. Nanomaterial-mediated RNA silencing offers a precise means of inhibiting gene expression, thereby reducing leukocyte recruitment without broadly impairing innate immune function [206]. For instance, siRNA-based silencing of endothelial adhesion molecules, including E-selectin, P-selectin, VCAM-1, ICAM-1, and ICAM-2, effectively decreases monocyte and neutrophil infiltration at lesion sites [177]. Beyond modulating endothelial activation, strategies targeting circulating immune cells have also shown promise. Pitavastatin-loaded NPs (pitavastatin-NPs), selectively internalized by monocytes/macrophages following intravenous administration, suppress NF-κB activation and downregulate MCP-1 and MMPs, thereby inhibiting monocyte chemotaxis via the MCP-1/CCR2 axis [178]. Additionally, pitavastatin-NPs may impair monocyte migration by inhibiting RhoA farnesylation independently of CCR2 expression. NP delivery of siTNF-α within liposomes further illustrates the cross-talk between TNF-α and MCP-1/CCL2. TNF-α silencing reduces MCP-1 production, thereby restricting macrophage recruitment [207]. Other chemokines, including CCL2, CCL5, CCL8, and CXCL9, have likewise emerged as potential nanotherapeutic targets [179].

In addition to suppressing chemokine production or receptor signaling, nanomaterials can directly sequester chemokines, neutralizing their effects and blocking immune cell recruitment. A notable example is the design of multifunctional immunosuppressive NPs (VINs), cloaked with membranes derived from mesenchymal stem cells (MSCs) engineered to overexpress CXCR4 [179]. This membrane camouflage provides immune evasion and homing capacity (“Trojan horse” or “cell hitchhiking”), enhancing NP accumulation at ischemic cardiovascular lesions via the CXCR4-CXCL12 axis. Moreover, the CXCR4-enriched surface of VINs acts as a “nano-decoy” that adsorbs and neutralizes CXCL12, thereby blocking infiltration of neutrophils and monocytes/macrophages into lesions. Importantly, the VIN core contains A151, a cyclic GMP-AMP synthase (cGAS) inhibitor, which suppresses cGAS-stimulator of interferon genes (STING) signaling in microglia and promotes M2 polarization, providing a synergistic dual anti-inflammatory effect.

Beyond therapy, immune cell chemotaxis itself provides valuable diagnostic opportunities. For instance, viral macrophage inflammatory protein-II (vMIP-II) has been exploited as a targeting ligand to generate a positron emission tomography (PET) tracer, ^64^Cu-vMIP-II-Comb. This comb-like peptide NP binds to CCRs CCR1, CCR2, and CXCR2 expressed on macrophages/monocytes, enabling sensitive and specific positron emission tomography/computed tomography (PET/CT) imaging of atherosclerotic lesions and other CVD-related inflammatory foci [180]. Such advances highlight the potential of chemokine- and CCR-targeted nanomaterials not only in therapeutic modulation but also in precise, noninvasive diagnosis of CVD progression.

### The regulation of immune cell proliferation

Immune cell proliferation is a key driver of CVD progression. Once activated, immune cells expand within cardiovascular lesions, perpetuating inflammation through sustained secretion of pro-inflammatory cytokines. Thus, localized suppression of aberrant immune cell proliferation has emerged as a promising therapeutic strategy to restrain disease progression [208, 209]. Nanomaterials, by delivering regulatory agents with spatiotemporal precision, can effectively block immune cell activation and proliferation during the early stages of inflammation, thereby mitigating downstream pathology [135, 210, 211].

One important approach is to prevent macrophage adhesion and subsequent activation at endothelial interfaces [184, 212, 213]. Rapamycin, a widely used immunosuppressant, exerts its effects primarily through inhibition of the mechanistic target of rapamycin complex 1 (mTORC1), a central regulator of cell growth, proliferation, motility, survival, and transcriptional activity [214, 215]. Recent advances in nanotechnology have facilitated the development of leukosomes, biomimetic nanovesicles generated by incorporating leukocyte membrane proteins into phospholipid bilayers [181]. Unlike rapamycin encapsulated in polymeric or inorganic carriers, rapamycin-loaded leukosomes (Leuko-Rapa) combine the physicochemical stability of nanomaterials with the biological properties of leukocytes, including immune evasion, prolonged circulation, and targeted delivery [216]. These features endow Leuko-Rapa with enhanced adhesion to ECs, enabling selective inhibition of NF-κB-mediated signaling in macrophages. Consequently, macrophage proliferation within lesion sites is reduced, alongside decreased production of TNF-α, IL-6, IL-1β, and chemotactic factors, thereby suppressing local inflammatory amplification. However, not all immune cell proliferation is detrimental. Treg expansion is protective, serving to restrain excessive inflammation and stabilize cardiovascular lesions. For example, active immunization with the autoantigen apolipoprotein B-100 (ApoB-100) peptide P210 has been shown to attenuate experimental AS. Chyu et al. [182] developed self-assembling amphipathic micelles (P210-PAM), which are taken up by DCs and subsequently promote the expansion of CD4^+^CD25^+^FOXP3^+^ Tregs and CD8^+^CTLA-4^+^ T cells. Expanded Tregs selectively modulate monocyte activity, skewing them toward less inflammatory phenotypes. This dual regulation of innate and adaptive immunity decreases lesion inflammation, underscoring the therapeutic value of selectively enhancing Treg proliferation in CVDs.

### The regulation of immune cell death mode

Distinct modes of immune cell death, including apoptosis, pyroptosis, necroptosis, and ferroptosis, differentially shape the inflammatory microenvironment and consequently influence the progression of CVDs. Apoptosis is generally considered immunologically silent, promoting resolution of inflammation and facilitating efferocytosis [217–220]. In contrast, pyroptosis and other lytic forms of cell death result in plasma membrane rupture and uncontrolled release of pro-inflammatory mediators, amplifying both local and systemic inflammation [221–224]. Thus, nanomaterial-based regulation of immune cell death has emerged as a promising therapeutic strategy to rebalance immune responses and suppress maladaptive inflammation in CVDs.

One effective therapeutic approach is to induce apoptosis of hyperactivated inflammatory immune cells, thereby controlling local inflammation and limiting collateral tissue damage. Kim et al. [202] designed H_2_O_2_-responsive poly (lactic-co-glycolic acid (PLGA) NPs (RC NPs) encapsulating catalase and peroxidase. Once internalized by neutrophils, catalase-mediated oxygen generation caused rapid NP bursting, releasing roscovitine (a cyclin-dependent kinase inhibitor) directly into neutrophils. This intervention redirected neutrophils from NETosis toward apoptosis, as indicated by the reduction of CitH3^+^ neutrophils. Neutrophil apoptosis induced by RC NPs promoted the recruitment and M2 polarization of cardiac macrophages. Through neutrophil-macrophage crosstalk, this strategy protected cardiomyocytes (CMs) and cardiac ECs from neutrophil-mediated injury and effectively preserved cardiac function. These findings highlight how nanomaterials can actively reprogram cell death pathways to convert pro-inflammatory cell fates into reparative ones.

Another promising paradigm is to inhibit pyroptosis, a lytic form of programmed cell death mediated by the NLRP3-caspase-1-IL-1β/IL-18 axis. Pyroptosis serves as a potent amplifier of inflammatory cascades, releasing IL-1β and IL-18 and triggering robust inflammatory responses in CVDs [225]. Targeting pyroptosis through nanotechnology, therefore, represents an attractive therapeutic avenue. For instance, Xiong et al. [226] targeted interferon regulatory factor 1 (IRF1), a master regulator of inflammation and cell death, using siRNA (siIRF1). Delivery of siIRF1 into M1 macrophages suppressed IFN-γ-mediated pyroptosis, thereby dampening downstream inflammatory cascades. In another study, neutrophil membrane-coated NPs were engineered to deliver puerarin (PU), a natural anti-apoptotic and antioxidative compound [227]. This biomimetic strategy exploited neutrophil homing properties to target inflamed cardiac tissues, while PU disrupted the detrimental crosstalk between macrophages and pyroptotic CMs. Mechanistically, it inhibited CMs pyroptosis through the NLRP3-caspase-1-IL-1β/IL-18 signaling pathway. Furthermore, reprogramming macrophages toward an M2 reparative phenotype enhanced the anti-pyroptotic effect by reshaping the inflammatory microenvironment, collectively attenuating myocardial injury.

These findings underscore the therapeutic promise of nanomaterial-mediated regulation of immune cell death modes. By inducing apoptosis in pathogenic neutrophils and macrophages while suppressing pyroptosis in CMs and inflammatory macrophages, nanomaterials provide a dual strategy to attenuate tissue-destructive inflammation and promote repair. A combinatorial approach targeting multiple cell death pathways in a stage- and cell type-specific manner may represent the next frontier for precision immunotherapy in CVDs.

### The regulation of immune cell function

#### Inhibition of excessive inflammatory response

Excessive activation of immune-mediated inflammation arises from the convergence of multiple pro-inflammatory cascades, with the NF-κB signaling pathway serving as a prototypical regulator [228]. NF-κB governs the transcription of numerous pro-inflammatory mediators across diverse innate immune cell subsets, making it a critical therapeutic target. Pharmacological inhibition of NF-κB signaling has thus emerged as a viable approach to suppress inflammatory cascades in CVDs. For example, Kim et al. [183] developed an exosome-based delivery system carrying a super-repressor IκB (Exo-srIκB), which effectively blocks NF-κB nuclear translocation and activation in neutrophils. Exo-srIκB reduces pro-inflammatory cytokine and chemokine expression, limits immune cell infiltration and adhesion molecule upregulation, and decreases apoptosis. Thus, these effects mitigate macrophage accumulation within lesions and attenuate ischemia-reperfusion-induced tissue injury. Similarly, siRNA-mediated silencing strategies can downregulate NF-κB signaling. Nanogels encapsulating siNF-κB reduce NF-κB p65 expression and lower downstream pro-inflammatory mediators [184]. In another example, macrophage membrane-camouflaged Apelin-13 NPs (MM/ANPs) suppress local inflammation and oxidative stress by concurrently inhibiting NF-κB and the NLRP3 inflammasome [185]. Targeting neutrophil-derived alarmins also represents a promising direction. Inhibition of S100A8/A9 by ABR2575 disrupts the S100A8/A9-NLRP3-IL-1β axis, attenuating neutrophil activation, clearing ROS, and limiting inflammatory amplification [186]. Moreover, nucleic acid-based nanotherapeutics, including si-Baf60a, pAnti-miR-33 [229], miR-146a/-181b [230], and DNAzyme silencing TNF-α [231], have demonstrated the capacity to fine-tune inflammatory pathways at the genetic level.

Another major therapeutic axis involves targeting ROS, which act as amplifiers of inflammation and mediators of tissue injury. He et al. [187] designed 2D black phosphorus nanosheets (BPNSs) functionalized with polyethylene glycol (PEG) and S2P peptides to load Resolvin D1 (RvD1), an inflammation-resolving lipid mediator (BPNSs@PEG-S2P/R). The S2P peptides confer macrophage-targeting capability, enabling preferential accumulation within lesions. Mechanistically, BPNSs scavenge ROS to restore macrophage oxidative phosphorylation and fatty acid oxidation, while RvD1 simultaneously resolves local inflammation. This dual antioxidant and anti-inflammatory effect effectively prevent AS progression. In addition to synthetic nanomaterials, enzyme mimetics targeting ROS detoxification pathways (e.g., superoxide dismutase [232], catalase [233], and peroxidase [234, 235]) have also shown potential in suppressing oxidative stress-driven inflammation. However, the rapid clearance of NPs by the reticuloendothelial system (RES) and the mononuclear phagocyte system (MPS) poses a major limitation for their therapeutic efficacy [236, 237]. To address this, biomimetic nanocarriers such as MM-coated NPs (MM-NPs) have been developed. These systems leverage natural cell-membrane properties to evade RES clearance, exploit immune self-recognition, and achieve lesion-specific accumulation. Moreover, MM-NPs can directly respond to inflammatory cues and pathogen-associated signals (e.g., bacterial toxins, viral particles, inflammatory cytokines), further enhancing their targeting specificity and therapeutic potential [188, 238].

Beyond targeting pro-inflammatory cascades and oxidative stress, nanomaterials can also harness the immune system’s endogenous homeostatic checkpoints. A representative example involves Tregs, which enforce immunotolerance and prevent immune overactivation. Liposomal NPs co-delivering MI-related antigens and rapamycin (L-Ag/R) were shown to induce tolerogenic DCs (tDCs) and antigen-specific Tregs, thereby establishing local immune tolerance, alleviating post-infarction inflammation, and promoting repair [189]. This strategy illustrates a host-directed nanotechnology approach that engages intrinsic regulatory circuits to dampen pathological immune hyperactivation while preserving essential defense functions.

#### Regulation of immune cell phagocytosis

Phagocytosis by innate immune cells plays a pivotal role in CVDs, with dual and contrasting implications. On the one hand, lipid phagocytosis by macrophages results in FCs formation, a hallmark of AS and a driver of panvascular disease progression [239]. On the other hand, efferocytosis (the clearance of apoptotic cells by healthy macrophages) prevents secondary necrosis and the release of pro-inflammatory intracellular contents, thereby suppressing inflammation, limiting necrotic lipid core expansion, and slowing disease progression [240]. These divergent outcomes make phagocytic regulation a promising therapeutic target for nanomaterial-based interventions.

A central checkpoint in efferocytosis is CD47, which functions as a “don’t-eat-me” signal on apoptotic and injured cells. Overexpression of CD47 on damaged myocardial cells in myocarditis, for instance, impedes macrophage-mediated clearance of dead cells and delays cardiac repair [241]. To overcome CD47-mediated inhibition of efferocytosis, Song et al. [190] encapsulated ferrostatin-1 and anti-CD47 antibodies within hollow mesoporous silica nanoparticles cloaked with platelet membranes. This biomimetic platform restored efferocytosis while simultaneously modulating ferroptosis, thereby reducing iron-dependent lipid peroxidation and its detrimental effects in CVDs. However, CD47 blockade alone may induce excessive cholesterol accumulation in phagocytes, leading to free cholesterol pooling and cholesterol crystal formation. To overcome this, Chuang et al. [191] engineered chimeric antigen receptor (CAR)-transduced THP-1 monocytes/macrophages (CAR-M) conjugated with β-cyclodextrin lipid NPs (β-CD LNPs), generating CAR-M/β-CD LNPs. This dual-modification system enhanced the selective clearance of CD47^high^ apoptotic cells within atherosclerotic lesions. Moreover, ROS-responsive disassembly of β-CD LNPs facilitated cholesterol processing: β-CD-mediated cholesterol oxidation produced oxysterols that activated liver X receptor (LXR) signaling, upregulating ATP-binding cassette protein A1 (ABCA1), ABCG1, and MerTK. These transcriptional changes enhanced cholesterol efflux while attenuating local inflammatory responses, thereby restoring phagocyte homeostasis.

A second major aspect of phagocytic regulation in CVDs involves cholesterol efflux, a critical process in lipid metabolism. Key regulators include ABCA1, ABCG1, and scavenger receptors (SRs), all of which are attractive nanotherapeutic targets [242–244]. The pathological progression of AS is strongly influenced by miRNAs that regulate cholesterol homeostasis. For instance, Nguyen et al. [192] developed a chitosan NP (chNP) delivery platform to transport miRNAs into macrophages, thereby modulating cholesterol receptor expression. Among these, miR-206 and miR-223-loaded chNPs enhanced ABCA1 expression and promoted cholesterol efflux.

Targeting SRs provides another avenue for intervention. He et al. [194] co-delivered SR-A siRNA (to suppress LDL uptake) and an LXR ligand (LXR-L) (to stimulate efflux) via mannose-functionalized dendritic NPs (mDNPs). This 2-pronged approach reduced SR-A expression while increasing ABCA1/ABCG1 levels, significantly alleviating AS progression. Similarly, 1 cM macromolecules, through their selective hydrophobic and charge-based targeting of inflamed macrophages, partially blocked SR-A-mediated ox-LDL uptake. When combined with the intracellular LXR agonist GW3965, this approach further promoted ox-LDL efflux and improved cholesterol clearance [193]. Together, these findings highlight that regulating phagocytosis by restoring efferocytosis, overcoming anti-phagocytic checkpoints such as CD47, and enhancing cholesterol efflux represents a promising therapeutic avenue. Nanomaterial-based platforms offer unique opportunities to precisely modulate these processes, thereby rebalancing immune and metabolic functions within atherosclerotic and ischemic lesions.

#### Regulation of macrophage polarization

Nanomaterials can modulate macrophage polarization through multiple signaling pathways, including cGAS-STING, NF-κB, STAT3, and PI3K/Akt1/mechanistic target of rapamycin (mTOR), achieving phenotypic reprogramming via anti-inflammatory and antioxidant mechanisms. For instance, an ginsenoside (Rb1)/polydopamine (PDA) hydrogel combines the antioxidant capacity of PDA with the immunomodulatory function of ginsenoside Rb1. PDA scavenges free radicals, alleviating mitochondrial oxidative stress and reducing mitochondrial DNA (mtDNA) release, thereby suppressing cGAS-STING signaling [195]. Transmission electron microscopy confirmed that Rb1/PDA treatment preserved mitochondrial cristae integrity and stabilized cell membranes, underscoring its antioxidative effect. Concurrently, Rb1 reduces cGAS activity by impairing DNA recognition and binding, which not only blocks cGAS-STING activation but also promotes STAT6/peroxisome proliferator-activated receptor gamma (PPAR-γ) signaling in M0 macrophages. This dual action facilitates IL-4 secretion, drives M2 polarization, and enhances the release of anti-inflammatory mediators. In vivo, Rb1/PDA hydrogels markedly reduced M1 macrophages and increased M2 macrophage populations within lesion areas, findings corroborated by RNA sequencing that revealed broad downregulation of oxidative stress- and immunity-related genes. Functionally, these polarization shifts improved CMs mitochondrial function, increased myocardial wall thickness, enhanced cardiac performance, and promoted myocardial regeneration after MI. Similarly, MNPs/Alg hydrogels promoted M2 differentiation via the PI3K/Akt1/mTOR pathway, elevating anti-inflammatory cytokine levels such as TGF-β, IL-10, and Arg1 [196].

By reprogramming macrophages from pro-inflammatory M1 toward reparative M2 phenotypes, nanomaterials indirectly promote tissue repair and angiogenesis. This shift not only suppresses destructive factors such as MMPs but also upregulates regenerative mediators including VEGF. For example, fibrinogen-loaded M@A-F/E NCs enhanced VEGF production and capillary network formation, significantly alleviating IRI in a transient middle cerebral artery occlusion (tMCAO) model [197]. The use of MM camouflage further improved delivery by enabling NPs to cross the blood-brain barrier (BBB), target ischemic infarcts, attenuate oxidative stress, promote microglial M2 polarization, restore neuronal mitochondrial membrane potential, reduce calcium overload, and remodel the inflammatory microenvironment. Other nanomaterials exploit cell-cell interactions to modulate macrophage polarization. Legume-derived ferulic acid peptide nanofibers (LFPNs) enhance macrophage-ECs crosstalk in inflamed lesions, promoting M2 polarization. M2-secreted cytokines such as TGF-β increased ECs adhesion and migration, while elevated VEGF-A and fibroblast growth factor (FGF)-2 secretion from macrophages led to enhanced angiogenesis, supported by higher CD31 and α-smooth muscle actin (α-SMA) expression at injury sites [198]. In addition, EVMS@NCs attenuated inflammatory cascades, preserved SMC contractile function, and reduced ECM degradation by lowering MMP-2 and MMP-9 expression, thereby mitigating vascular remodeling [199]. These studies highlight the central role of nanomaterials in macrophage polarization therapy: by suppressing M1-driven inflammation and enhancing M2-mediated repair, nanomaterials provide a dual advantage of dampening destructive immune responses while simultaneously promoting tissue regeneration and angiogenesis. This dual regulatory capacity makes macrophage polarization a particularly attractive target in nanomedicine-based therapies for CVDs.

#### Regulation of NETs formation

Neutrophils critically contribute to the progression of CVDs through the formation of NETs. NETs are generated via a specialized form of programmed cell death termed NETosis, during which neutrophils release chromatin fibers decorated with histones (e.g., H3Cit) and granular proteins (e.g., MPO, NE). These structures activate ECs and perpetuate vascular inflammation [245, 246]. Thus, inhibiting NETosis and preventing excessive NET formation has emerged as a promising therapeutic strategy in CVDs.

One approach involves targeting peptidyl arginine deiminase 4 (PAD4), an essential enzyme for NETosis. Col IV-NPs encapsulating the PAD4 inhibitor GSK484 selectively accumulate at vascular sites where ECs are disrupted and the collagen IV-rich basement membrane is exposed. This localized delivery efficiently blocks PAD4 activity, thereby reducing NETs production [200]. Functionally, this intervention improved endothelial continuity and markedly reduced NETs accumulation at intimal lesion sites. Interestingly, the physicochemical properties of NPs themselves influence NET formation. Particles in the 10–40 nm range can disrupt plasma membranes and destabilize lysosomes, triggering rapid NETosis. In contrast, larger NPs (100–1000 nm) cause minimal membrane perturbation and exhibit substantially reduced pro-NETotic activity [247–249]. Superparamagnetic iron oxide NPs (SPIONs, SPION^LA1^, and SPION^LA2^) further demonstrated that under magnetic field exposure, their topological changes promoted NET formation and thrombogenesis [250]. However, coating SPIONs with human serum albumin (SPION^LA−HSA^) or dextran (SPION^DEX^) significantly inhibited this process, preventing irreversible NET aggregates and thereby reducing thrombosis and vascular occlusion. These findings highlight that rational NP size and surface design can mitigate the risk of NET-driven pathology.

Another therapeutic strategy is the enzymatic degradation of NETs. DNase I, which cleaves extracellular DNA, has shown efficacy in dismantling NET structures, thereby alleviating their pathogenic consequences in vascular lesions [251]. Beyond direct NET inhibition, nanomaterials can also indirectly suppress NETosis by attenuating oxidative stress and mitochondrial dysfunction. Gong et al. [201] designed a PtIr nanozyme with intrinsic antioxidative activity that enhances mitochondrial function, lowers ROS levels, reduces apoptosis, and ultimately suppresses NET formation. In vivo, this intervention increased microvascular density, decreased fibrosis, restored perfusion, and improved long-term cardiac function, thereby offering protection to CMs.

These findings underscore that regulating NET formation, whether by enzyme inhibition, NP design, enzymatic degradation, or redox modulation, represents a critical therapeutic avenue. Nanomaterials, by leveraging these mechanisms, provide powerful and precise strategies to attenuate NET-driven inflammation, thrombosis, and tissue damage in CVDs.

## Nanomaterials enhance imaging in CVDs

The degree of local inflammation in CVDs is closely linked to the burden and activity of immune cells, which are critical drivers of disease initiation and progression [25]. Thus, strategies that allow real-time visualization of immune cell distribution and activity within cardiovascular lesions are invaluable for both disease monitoring and treatment guidance. By coupling adjustable physicochemical properties with tailored interactions at immune cell and endothelial interfaces, nanoengineered probes have become potent agents for cardiovascular immune imaging [82, 252–254]. When internalized by immune cells or accumulated in inflamed tissues, nanomaterials generate distinct imaging signals that correlate with lesion severity, thereby enabling precise assessment of disease status.

Imaging modalities has been employed in nanomaterial-based cardiovascular diagnostics, each offering complementary advantages. These include fluorescence imaging (FLI) for high sensitivity, magnetic resonance imaging (MRI) for superior anatomical resolution, photoacoustic imaging (PAI) for deep-tissue vascular visualization, ultrasound for dynamic and noninvasive functional assessment, CT for structural analysis, and radionuclide imaging techniques such as PET and single-photon emission computed tomography (SPECT), which provide highly sensitive quantitative detection. Furthermore, multimodal imaging approaches, such as PET/CT and PAI/MRI, combine anatomical and functional information, offering more comprehensive insights into CVD pathophysiology and treatment responses [255].

A key mechanism enabling nanomaterial-based lesion visualization is their selective accumulation within inflamed cardiovascular tissues. This occurs either through interactions with immune cells (e.g., macrophages, neutrophils, and T cells recruited to lesions) or via binding to specific molecular targets in the inflammatory microenvironment, such as adhesion molecules, chemokines, or ECM components (Table 2) [256–262]. These targeting strategies enhance imaging specificity, allowing detection of otherwise occult inflammatory changes, delineation of plaque vulnerability, and monitoring of microvascular remodeling. Beyond diagnosis, nanomaterials integrated into imaging systems also facilitate image-guided therapeutic interventions. For example, theranostic nanoplatforms can simultaneously deliver therapeutic agents (e.g., anti-inflammatory drugs, siRNAs, or antioxidants) while enabling real-time imaging of biodistribution and treatment efficacy [263–266]. Such platforms support dynamic monitoring of lesion regression, evaluation of immune modulation, and optimization of therapeutic regimens, thereby accelerating translation into precision cardiovascular medicine.

**Table 2:** Representative nanodiagnostic imaging materials in CVDs

Nanomaterials	Target sites	Class of nanomaterials	Size of nanomaterials	Imaging modality	Imaging agent	Target composition	Targeting mechanism	Imaging advantages	References
^18^F-DBCOT-MSN-RAW	Targeting immune cells	Inorganic nanomaterials	N/A	PET	^18^F	Macrophages	Targeting through macrophage homing	The AS plaque region exhibited higher SUV and PET signals. And it makes ^18^F exhibit a longer half-life	[256]
Macroflor	Targeting immune cells	Polymers	(5.0 ± 0.4) nm	PET	^18^F	Macrins	N/A	Macrophages in the AS and MI regions showed significantly increased selective uptake of Macroflor and almost negligible uptake by CMs, reducing false-positive signals	[257]
anti-MARCO UCNPs	Targeting immune cells	Inorganic nanomaterials	(26.7 ± 0.8) nm	NIRF, MRI	NaGdF_4_: Yb, Er@NaGdF_4_	MARCO antibody	MARCO antibodies can target and bind MARCO on the surface of M1 macrophages	Anti-MARCO UCNPs selectively target M1 macrophages to recognize vulnerable plaques	[258]
^89^Zr-AI-HDL ^89^Zr-PL-HDL	Targeting immune cells	Inorganic nanomaterials	(8.6 ± 0.9) nm (8.2 ± 0.9) nm	PET, CT, MRI	^89^Zr	HDL	HDL can target proteins on macrophages, such as ABCG1	Different ^89^Zr labeling positions enable the study of the biological behavior of HDL in vivo	[259]
PEG-*bl*-PPS PS	Targeting immune cells	Polymers	113.7 nm	NIRF	ICG	PEG-*bl*-PPS PS	N/A	It was found that superior targeting of DCs can be achieved by changing the structural morphology of nanomaterials, even when the targeting ligand is not modified	[260]
MM@Ce-CDs NPs	Targeting the inflammatory microenvironment	Inorganic nanomaterials	166.65 nm	PAI, FLI	CDs	MM	MM surface integrin α4β1 specifically binds to overexpressed VCAM-1	The PA and FL properties of Ce-CDs were selectively activated by taking full advantage of the high ROS in plaques, which realized the targeting of AS while completing the removal of ROS	[261]
DMM	Targeting the inflammatory microenvironment	Inorganic nanomaterials	300 nm	MRI	Mn^2+^	N/A	N/A	The acidic microenvironment in the corresponding plaques of DMM releases Mn^2+^ for MRI imaging, which has a long imaging window	[262]

*MSN* mesoporous silica nanoparticle, ^*18*^*F-DBCOT-MSNs* ^18^F-labeled aza-dibenzocycloocta-triazolic MSNs, *N/A* not applicable, *PET* positron emission tomography, *AS* atherosclerosis, *SUV* standardized uptake value, *MI* myocardial infarction, *CMs* cardiomyocytes, *MARCO* macrophage receptor with collagenous structure, *UCNPs* upconversion nanoparticles, *NIRF* near-infrared fluorescence, *MRI* magnetic resonance imaging, *M1* classically activated macrophage phenotype, *HDL* high-density lipoprotein, ^*89*^*Zr-AI-HDL* ^89^Zr-apolipoprotein A-I-labeled HDL, ^*89*^*Zr-PL-HDL* ^89^Zr-phospholipid-labeled HDL, *CT* computed tomography, *ABCG1* ATP-binding cassette subfamily G member 1, *PEG-bl-PPS PS* poly(ethylene glycol)-bl-poly(propylene sulfide) polymersomes, *ICG* indocyanine green, *DCs* dendritic cells, *NPs* nanoparticles, *PAI* photoacoustic imaging, *FLI* fluorescence imaging, *CDs* carbon dots, *MM* macrophage membrane,*VCAM-1* vascular cell adhesion molecule-1, *DMM* diallyl trisulfide-loaded metal-organic cage-68-doped poly(ethylene glycol)-modified manganese dioxide nanoparticles

### Targeting immune cells at lesion sites

The expression of distinct surface molecules on immune cells provides unique opportunities for molecularly targeted imaging in CVDs. Among immune cells, macrophages have been the predominant focus due to their central roles in lesion initiation, progression, and resolution. Macrophages express a wide range of surface markers, including CD40, CD86, integrin-α4β1, integrin-αMβ2, CCR2, phosphatidylserine, and OPN, that can be exploited for selective targeting by nanomaterials [267–271]. In addition to macrophages, DCs also accumulate within atherosclerotic plaques and influence immune balance, representing another promising target for nanomaterial-based imaging approaches [157, 272, 273].

During the early phases of AS, circulating immune cells are recruited to lesion sites, making their tracking a powerful approach for evaluating therapeutic responses and characterizing disease progression at the molecular level. The PET radiotracer ^18^F-fluorodeoxyglucose (^18^F-FDG), widely internalized by metabolically active macrophages, has been extensively used to visualize inflamed plaques [274]. However, its relatively short half-life (t_1/2_ = 109.8 min) limits long-term monitoring in PET-based cell tracking [275]. To overcome this, azide-dibenzocyclooctyne (DBCO)-functionalized mesoporous silica NPs (MSNs) were engineered to prolong the half-life of ^18^F [256]. Following co-incubation with RAW 264.7 macrophages, these NPs formed DBCO-MSN-RAW complexes, which were subsequently radiolabeled via strain-promoted azide-alkyne cycloaddition (SPAAC) to yield ^18^F-DBCO-MSN-RAW cells. PET imaging revealed robust macrophage migration signals at plaque sites, with elevated signal intensity detectable as early as 1 h post-injection and persisting for up to 8 d. Importantly, reduced macrophage accumulation correlated with lesion regression, underscoring the dual value of this strategy for both short-term tracking of immune cell trafficking and long-term evaluation of plaque burden. Despite these advantages, ^18^F-FDG exhibits limited specificity due to high myocardial uptake, increasing the risk of false positives [276]. To address this, Keliher et al. [257] developed Macroflor, a PET imaging agent based on ^18^F-labeled 5 nm polyglucose NPs. Macroflor demonstrated high macrophage affinity and strong lesion-specific accumulation in both AS and MI models. Unlike ^18^F-FDG, Macroflor exhibited negligible CM uptake, achieving superior specificity and favorable pharmacokinetics. Although limited by its short half-life, Macroflor remains a promising agent for macrophage-targeted cardiovascular imaging.

Beyond total macrophage burden, nanomaterials also enable monitoring of macrophage polarization states, which serve as indicators of local inflammatory activity and plaque vulnerability. Wang et al. [258] developed anti-MARCO-modified NaGdF_4_: Yb, Er@NaGdF_4_ upconversion NPs (anti-MARCO UCNPs) to specifically track M1 macrophages. MARCO, a receptor selectively upregulated in M1 macrophages and ox-LDL-induced FCs, was exploited for targeted imaging. FLI and MRI demonstrated significantly enhanced signals in rupture-prone plaques, distinguishing them from stable lesions. Notably, FLI provided superior sensitivity and real-time monitoring capability, allowing dynamic evaluation of M1 macrophage activity at lesion sites and facilitating early assessment of plaque destabilization.

In addition to synthetic nanomaterials, endogenous NPs such as HDL have been leveraged as natural carriers for immune cell-targeted imaging. HDL exhibits intrinsic tropism for macrophages via interactions with lipid transporters such as ABCG1. Pérez-Medina et al*.* [259] demonstrated that radiolabeled HDL NPs preferentially accumulated in macrophage-rich plaques, irrespective of the labeling position. In atherosclerotic mice, ^89^Zr-labeled HDL exhibited strong plaque-associated signals, with prolonged circulation (half-life = 1.13 d) confirmed in rabbit models, reflecting impaired lipid metabolism and clearance in AS. Moreover, DCs also persist throughout all stages of AS and have been implicated in both pro- and anti-atherogenic processes [277]. However, strategies for real-time monitoring of DC behavior remain limited. Near-infrared fluorescent (NIRF) imaging has emerged as a promising technique, enabling visualization of DC spatial distribution within plaques and characterization of their uptake of engineered nanomaterials [260]. Such advances may provide critical insights into DC-driven immunopathology and guide the development of DC-targeted theranostic approaches in panvascular diseases.

### Targeting the inflammatory microenvironment in CVDs

The inflammatory microenvironment of atherosclerotic plaques is characterized by excessive oxidative stress and metabolic reprogramming. Lipid peroxidation and inflammatory activation signals from FCs derived from ECs, macrophages, and VSMCs stimulate the upregulation of NADPH oxidase, leading to increased ROS production. Elevated ROS levels establish a self-perpetuating positive feedback loop that enhances immune cell recruitment and accelerates inflammatory infiltration within plaques [60, 278]. Meanwhile, persistent oxidative stress and immune activation drive glycolytic shifts in macrophages, ECs, and VSMCs, causing lactate accumulation and intraplaque acidosis [279–281]. Collectively, this results in a unique plaque microenvironment distinguished by high ROS levels and mildly acidic pH, which can be exploited as dual stimuli for the design of nanomaterial-based imaging and theranostic platforms.

Aberrant oxidative stress in plaques provides a powerful biochemical cue for nanomaterial activation. Carbon dots (CDs), owing to their exceptional PA and NIRF properties, have been adapted for ROS-responsive imaging. For example, cerium-doped CDs (Ce-CDs) coated with MM achieve inflammation-specific targeting via VCAM-1/integrin-α4β1 interactions with activated ECs [261]. Under physiological conditions, Ce-CDs maintain quenched fluorescence and PA signals; however, ROS-rich plaque microenvironments activate Ce^3+^/Ce^4+^ redox cycling, selectively restoring these signals and enabling precise PAI of plaque inflammatory status. MM cloaking further enhances homotypic targeting, evidenced by markedly stronger fluorescence signals in atherosclerotic lesions compared to controls. This approach provides a powerful platform for inflammation mapping, vulnerable plaque identification, and post-therapy monitoring in AS.

The acidic milieu of plaques also represents a valuable trigger for responsive nanomaterials. Li et al. [262] developed dual-gas-generating theranostic NPs by encapsulating diallyl trisulfide (DATS) into MOC-68-doped PEGylated MnO_2_ nanocarriers (DATS ⊂ MOC-68@MnO_2_@PEG, abbreviated DMM). The hollow mesoporous MnO_2_ core is highly responsive to low pH and H_2_O_2_, generating O_2_ in situ to relieve hypoxia while simultaneously releasing Mn^2+^ ions for MRI. The released Mn^2+^ exhibited superior longitudinal relaxivity, surpassing that of conventional clinical contrast agents such as gadopentetate dimeglumine. In vivo, DMM enabled rapid T1-weighted contrast enhancement of aortic plaques within 2 h, peaking at 12 h and persisting for up to 48 h. T1 mapping confirmed progressive signal attenuation corresponding to Mn^2+^ release, validating the responsiveness of this system to plaque microenvironments. Such prolonged retention not only enhances diagnostic accuracy but also reduces dosing frequency, improving clinical safety.

Beyond ROS and acidity, MMPs, particularly MMP-12, are abundantly expressed within plaques and correlate with lesion instability. MMP-12 represents an additional molecular target for nanomaterial functionalization, offering opportunities to design enzyme-activated nanosystems for lesion detection and targeted therapy [282]. Expanding this strategy to other microenvironmental hallmarks, such as hypoxia, altered lipid metabolism, and inflammatory cytokine gradients, could further improve the precision and specificity of nanotheranostic platforms in CVDs.

## Nanomaterials in cardiovascular disease treatment by regulating immune cell functions

### Treatment of panvascular disease

Panvascular disease is a systemic vascular disorder characterized by widespread pathological changes across multiple vascular beds, with AS accounting for approximately 95% of cases [6, 283]. Clinically, it may manifest as distinct vascular pathologies, including coronary artery disease, cerebrovascular lesions, and peripheral arterial disease, or as concurrent involvement of multiple circulatory territories [6]. Among these, AS constitutes the principal pathological hallmark. The progression of AS is a systemic and continuous process that affects the entire arterial system. Early stages are characterized by lipid deposition, immune cell infiltration, and inflammatory activation, which together promote the formation of atherosclerotic plaques. As plaques enlarge and destabilize, they may undergo erosion or rupture, leading to superimposed thrombosis. These events represent the primary pathological basis for acute ischemic syndromes, including acute MI, IS, and severe limb ischemia [284, 285]. Conversely, some unstable plaques undergo healing and fibrous remodeling, transforming into collagen-rich stable plaques that are less prone to rupture [286]. This transformation is associated with improved outcomes and provides a therapeutic window for systemic management of CVDs.

The cornerstone of panvascular disease management involves anti-inflammatory, antithrombotic, and lipid-lowering therapies, which collectively aim to stabilize plaques, prevent rupture, and reduce ischemic events. Notably, these therapeutic goals align closely with the mechanisms by which nanomaterials regulate immune cell behavior [82, 287, 288]. By modulating inflammation, suppressing prothrombotic signaling, and restoring lipid metabolic balance, nanomaterial-based interventions hold promise in halting AS progression and thereby ameliorating downstream pathologies across multiple vascular territories [289–292]. This convergence highlights the potential of nanomedicine to serve not only as a local therapeutic modality but also as a systemic strategy for the precision management of panvascular diseases.

#### Treatment of AS

The initiation of AS is marked by the recruitment of immune cells to lesion sites under the influence of chemokines, followed by interactions with vascular ECs mediated by adhesion molecules, enabling monocyte and neutrophil transmigration into the intima. Nanomaterials can effectively interrupt these early events. For instance, TRAF-STOPs, developed by Seijkens et al. [293], are low-molecular-weight compounds that selectively attenuate CD40-CD40 ligand (CD40L) signaling in monocytic lineages by disrupting CD40-TNF receptor-associated factor 6 (TRAF6) interactions. This results in reduced phosphorylation of NF-κB intermediates, thereby suppressing macrophage recruitment, activation, and downstream vascular inflammation. Similarly, neutrophil membrane-camouflaged nanoplatforms (AM@ZIF@NM) were engineered to deliver antisense oligonucleotides (ASOs) against microRNA-155 to ECs, reducing miR-155 levels and restoring expression of its target gene BCL. By inhibiting the NF-κB pathway, this strategy decreased *RELA*, *ICAM-1*, and *CCL2* expression, suppressed vascular inflammation, and prevented VSMC proliferation and collagen deposition, collectively hindering AS progression [294].

Macrophage lipid metabolism plays a central role in plaque development. Internalization of apolipoprotein B-containing lipoproteins (apoB-LPs) by macrophages drives FC formation, triggering endoplasmic reticulum stress, apoptosis, and release of MMPs, thereby exacerbating necrotic core expansion and plaque instability. Modulating macrophage lipid handling, therefore, represents a key therapeutic target. For example, Li et al. [295] used ASOs against miR-33 (anti-miR-33) in combination with cRGDfK peptide to form RAAM NPs, which enhanced integrin-mediated targeting. These NPs restored ABCA1/ABCG1 function, promoted cholesterol efflux, suppressed FC formation, and enhanced Treg (FoxP3^+^ Treg) infiltration within lesions, thereby coupling lipid regulation with adaptive immune protection against AS.

In addition, inflammation and oxidative stress within the plaque microenvironment are critical drivers of disease progression. Nanomaterials provide unique opportunities to modulate both. For instance, Liang et al. [234] engineered NeuM@P5c/S/C NPs by loading SOD and catalase (CAT) onto P5c, a carrier capable of efficient cytoplasmic protein delivery. This system scavenged ROS, alleviated oxidative stress, inhibited FC formation, and promoted M2 polarization, accompanied by reduced TNF-α and IL-6 expression. NeuM@P5c/S/C also enhanced autophagy, reduced DNA damage, and suppressed senescent cell burden within plaques, thereby thickening fibrous caps via MMP-9 inhibition. Other strategies include direct cytokine delivery: Kamaly et al. [296] designed Col IV-IL-10 NPs, which selectively targeted lesions, elevated pSTAT3, reduced necrotic cores, and promoted fibrous cap thickening without altering macrophage or SMC density. Similarly, Col IV-Ac2-26 NPs, targeting the formyl peptide receptor 2/ lipoxin A_4_ receptor (FPR2/ALX) receptor, upregulated IL-10 and TGF-β expression, increased collagen synthesis by SMCs, and promoted inflammation resolution [297]. Moreover, NLRP3 inflammasome inhibition by nanocarriers effectively reduced CD3^+^ T cell infiltration and plaque inflammation [298].

Another promising direction involves regulating macrophage efferocytosis, the process by which apoptotic cells are cleared. In AS, defective efferocytosis promotes necrotic core expansion. Targeted nanoplatforms such as S2P-siCamk2g NPs deliver siRNA against Ca^2+^/calmodulin-dependent protein kinase γ (CaMKIIγ), thereby reactivating the MerTK pathway in macrophages, repairing efferocytosis defects, clearing apoptotic FCs, and preventing fibrous cap rupture [299]. Plaque vulnerability is largely determined by fibrous cap integrity, which depends on the balance of collagen synthesis and degradation. Vulnerable plaques, with large necrotic cores and thin fibrous caps, are enriched in MMP activity, VSMC phenotypic switching, and persistent inflammatory infiltration [300, 301]. Macrophage-derived MMPs, especially MMP-1, MMP-8, and MMP-12, degrade collagen and elastin, promoting cap thinning and rupture [302–305]. Regulating macrophage polarization can reduce deleterious MMP release. For instance, Nakashiro et al*.* [235] used pioglitazone-loaded NPs to activate PPARγ, enhancing M2 macrophage polarization (CD36, CD206, Arg-1, IL-10) while suppressing pro-inflammatory mediators [IL-6, MMP-9, extracellular matrix metalloproteinase inducer (EMMPRIN)]. This strategy inhibited ECM degradation, stabilized plaques, and prevented rupture. Once a plaque ruptures, blood contacts the subendothelial matrix and necrotic core, triggering occlusive thrombus formation mediated by tissue factor, factor XIIa, VIIa, and thrombin [306, 307]. Nanomaterials can intervene by delivering thrombolytic or anticoagulant agents, including tissue plasminogen activator (tPA) [308–310], streptokinase [311, 312], urokinase [313], and direct thrombin inhibitors [314]. Although most nanomaterial applications in thrombosis have focused on diagnostic imaging, targeted immunomodulatory approaches to prevent immune cell-driven thrombosis remain underexplored.

AS plaques that narrow or occlude the lumen impair blood flow, leading to ischemia, hypoxia, or necrosis in downstream tissues. Coronary artery involvement results in MI, while cerebrovascular occlusion causes IS. Thus, treating AS not only stabilizes plaques but also serves as the foundation for systemic management of panvascular diseases. Importantly, given the heterogeneity of CVDs, specific conditions such as MI, IS, and peripheral arterial disease demand tailored nanomaterial-based interventions built on the shared pathophysiological basis of AS [6].

#### Treatment of MI

AS represents the fundamental pathological substrate of MI, and nanomaterial-based treatments for MI share mechanistic similarities with anti-atherosclerotic strategies. However, MI is distinguished by the extensive damage to CMs and the limited regenerative capacity of cardiac tissue. Consequently, post-infarct repair relies predominantly on scar formation, where immune cells play critical roles in orchestrating inflammation, fibrosis, and tissue remodeling.

Following MI, pro-inflammatory immune cells are rapidly recruited to the infarct zone, releasing cytokines such as TNF-α, IL-1β, and IL-6, which exacerbate inflammation while facilitating necrotic tissue clearance [315]. During the early pro-inflammatory phase, these mediators transiently inhibit fibroblast activity, whereas in the subsequent anti-inflammatory phase, cytokines including IL-10 and TGF-β promote fibroblast-to-myofibroblast differentiation, initiating reparative fibrosis [316, 317]. Therefore, a therapeutic focus of nanomaterials is to accelerate the transition toward anti-inflammatory phenotypes and promote scar stabilization. For instance, Gel@MSN/miR-21-5p, a pH-responsive delivery system, releases miR-21-5p specifically at acidic infarct sites [318]. Here, mesoporous silica NPs suppress the TLR2-NF-κB axis, reducing pro-inflammatory cytokine production, while miR-21-5p enhances VEGFA expression by targeting sprouty receptor tyrosine kinase signaling antagonist 1 (SPRY1), thereby promoting angiogenesis. Together, these effects mitigate fibrosis and improve post-MI cardiac remodeling. Similarly, poly-ε-caprolactone (PCL) /derivative dimethyl itaconate (DMI) nanofibers attenuate the IL-23/IL-17 pro-inflammatory cascade, upregulate nuclear factor erythroid 2-related factor 2 (Nrf2)-mediated antioxidant genes, and sustain M2 macrophage polarization (Arg-1, KLF-4, YM-1, CD206), ultimately reducing infarct size [319]. Another innovative approach leverages systemic immune regulation. eSENT NPs, loaded with histone deacetylase (HDAC) inhibitors, act via the heart-spleen axis to modulate splenic monocytes under myocardial injury conditions, significantly reducing infarct size by up to 33-fold at doses 14-fold lower than systemic administration. Notably, their activity at the border zone between infarct and viable myocardium highlights the therapeutic potential of regional immune modulation [320].

Post-MI hypoxia exacerbates tissue necrosis and impedes repair. To address this, Au@Pt/Alg hydrogels combined with brown adipose stem cells (BASCs) have been developed [321]. Pt NPs scavenge ROS, improving BASC survival and differentiation into CMs. In parallel, BASC-secreted VEGF promotes angiogenesis within infarcted myocardium. Importantly, Au@Pt/Alg hydrogels also enhance electrical signal propagation across scar tissue, enabling residual viable myocardium within scars to contract synchronously with surrounding healthy tissue, thereby improving cardiac output. Furthermore, ROS clearance upregulates connexin-43 (Cx43), enhancing intercellular electrical coupling and reducing CM apoptosis. Collectively, this strategy integrates antioxidative, angiogenic, and electrophysiological restoration, significantly improving functional recovery after MI.

Sudden reperfusion post-MI often induces IRI, characterized by ROS surges, immune hyperactivation, and pathological fibrosis [322]. Nanomaterials have shown promise in mitigating this damage. For example, MNM/siRNA NPs, engineered with hemagglutinin (HA) and integrin-functionalized neutrophil membranes, enable efficient siRNA delivery through endosomal escape while maintaining immune-targeting specificity [111]. Inhibition of integrin-α9 reduced neutrophil infiltration, NETosis (PAD4, MPO, CitH3), and cytokine release (TNF-α, IL-1β, IL-6), thereby alleviating microthrombosis and preserving endothelial integrity. In IRI mouse models, these NPs significantly reduced infarct size and improved cardiac function. Similarly, CsA@PPTK NPs, camouflaged with platelet membranes and loaded with cyclosporine A, enhanced Treg generation and M2 macrophage polarization while scavenging ROS [323]. This dual regulation decreased myocardial apoptosis, reduced fibrosis, and improved left ventricular remodeling. CsA@PPTK also downregulated MMP-9 while upregulating CX43 and α-SMA, supporting structural repair and contractile recovery.

Preconditioning strategies have also been investigated to enhance myocardial tolerance against ischemic insults. Liu et al*.* [324] designed a hydrogel-coated upconversion cyanobacteria nanocapsule (UCCy@Gel) that consumes oxygen via respiration, thereby generating a controlled hypoxic microenvironment and upregulating cardioprotective HSP70. Upon near-infrared (980 nm) irradiation, UCCy@Gel produced photosynthetic oxygen via upconversion luminescence (UCL), simultaneously suppressing M1 macrophage polarization and downregulating IL-6 and TNF-α, thereby facilitating myocardial repair. This approach illustrates how nanotechnology-assisted ischemic preconditioning can enhance cardioprotection by combining hypoxic adaptation with immune modulation.

#### Treatment of IS

IS, which accounts for over 80% of all strokes, is primarily caused by thrombosis and subsequent disruption of cerebral blood supply, making it a prototypical systemic manifestation of arteriosclerotic disease [325–327]. Consequently, thrombolytic therapy remains a cornerstone of treatment. Similar to MI, the reperfusion phase of IS can also induce IRI, characterized by sharp increases in ROS, which exacerbate neuronal injury and tissue remodeling [328–330]. Thus, ROS clearance has emerged as an effective therapeutic strategy to improve post-IS outcomes. Another defining feature of IS is the complex interplay of molecular and cellular events involving neuronal injury, BBB disruption, microglial and astrocyte activation, and neuroinflammation, making neuroprotection a central focus of therapy [331].

Accordingly, IS treatment strategies integrate thrombolysis, anti-inflammatory and anti-oxidative interventions, and neuroprotective approaches. For example, Kong et al. [332] designed a platelet-membrane (PLM)-camouflaged, ROS-responsive NP (rPZDCu) for targeted thrombolysis and antioxidation. This system encapsulated Cu_4.6_O NPs, an ultrasmall, non-stoichiometric copper oxide nanoparticle with mixed Cu/Cu_2_O valence, in zein-Se-Se-DHA (ZDCu), subsequently coated with PLMs and conjugated with rt-PA to generate rPZDCu. In the ROS-rich ischemic microenvironment, Cu₄.₆O and DHA were released, exhibiting potent ROS-scavenging activity and driving M1-to-M2 microglial polarization while downregulating TNF-α, IL-1β, and IL-6 [333]. The PLM coating enabled lesion-specific targeting and enhanced fibrinolytic activity. Histological analysis confirmed preserved neuronal morphology and effective neuroprotection, underscoring the potential of integrating ROS-responsive antioxidant delivery with thrombolytic therapy. Notably, The BBB remains a major barrier for nanomaterial delivery into ischemic brain regions. To overcome this, cell-hitchhiking strategies have been developed, exploiting neutrophils, which are the earliest and most abundant infiltrating cells at ischemic sites. One such platform, AP@R, leverages both active and passive targeting of neutrophils, which transport the therapeutic cargo across the BBB [334]. Once in the infarct zone, neutrophils disassemble to release cilostazol, which inhibits lipocalin-2 (Lcn2) expression in astrocytes, suppressing the A1 pro-inflammatory subtype while promoting A2 polarization. This shift was validated by reduced Lcn2 and C3 signals, alongside elevated S100A10 expression, indicating effective astrocytic reprogramming and neuroprotection. As Lcn2 drives neurotoxicity through A1 polarization, its suppression provides robust neuronal protection and mitigates long-term damage. Furthermore, Lcn2 downregulation reduced expression of the MMP9-Lcn2 complex and CXCL10, promoting BBB repair.

Other biomimetic strategies have also shown efficacy. For instance, M2-MM-coated NPs demonstrated strong tropism toward ischemia-activated brain microvascular ECs [335]. Immunofluorescence revealed specific co-localization with microglia in the ipsilateral hemisphere after IS. The M2 membranes provided anti-inflammatory cytokines that promoted microglial polarization toward M2 phenotypes, reduced MPO expression (reflecting decreased neutrophil infiltration), and attenuated neuronal apoptosis. The therapeutic effect of baicalin (BA) was further enhanced, as evidenced by downregulated B-cell lymphoma 2 (Bcl-2) and upregulated Bcl-2-associated X protein expression, confirming neuroprotective efficacy.

Beyond microglial and astrocytic modulation, targeting the inflammasome and signaling pathways offers additional opportunities. Inhibition of the absent in AIM2 inflammasome or MAPK signaling pathway has been demonstrated to suppress neuroinflammation, reduce infarct size, and improve neurological recovery in IS models [185, 336, 337]. These findings underscore the potential of integrating molecularly targeted nanotherapies with immunomodulatory and thrombolytic approaches for comprehensive IS management.

#### Treatment of abdominal aortic aneurysm (AAA)

Aneurysms occur most frequently in the aorta, with AAA representing the predominant subtype [338]. The principal pathological hallmarks of AAA include VSMC apoptosis, chronic inflammation, ECM degradation, and thrombus formation [339–341], collectively leading to irreversible, localized aortic dilation [342]. The pathogenesis of AAA is closely linked to the broader mechanisms of systemic vasculopathy, particularly the degradation of the elastic lamina in atherosclerotic vessels. Inflammatory cell infiltration, aberrant neovascularization, and dysregulated protease and cytokine cascades are central drivers of disease progression [343]. Thus, AAA treatment strategies can be conceptualized within the systemic therapeutic framework of panvascular disease.

Among immune cells, neutrophils play a central pathogenic role by releasing oxidative mediators, pro-inflammatory cytokines and chemokines, leukotrienes, and proteases (e.g., elastase, MMPs). These mediators promote intraluminal thrombus formation, stimulate monocyte recruitment, degrade elastin and ECM, and amplify adventitial inflammation, collectively exacerbating oxidative stress and vascular wall damage [344–346]. To counteract these effects, Hu et al. [347] developed a luminol-conjugated α-cyclodextrin material (LaCD) NPs, which effectively suppressed neutrophil infiltration, reduced neutrophil activation, and inhibited MPO release, thereby attenuating NET formation. LaCD NPs significantly downregulated aberrant *Mpo* gene expression within aneurysmal aortae. Mechanistically, LaCD NPs reduced NET-driven VSMC apoptosis, preserved VSMC viability, and attenuated aneurysmal dilation. Compared with PAD4 inhibitor GSK484, LaCD NPs demonstrated superior efficacy in preventing aortic expansion and limiting AAA progression.

Vascular calcification is another important risk factor for AAA rupture [348]. Pharmacological inhibition of this process represents an attractive therapeutic target. For example, rapamycin, a classical immunosuppressant, has been shown to limit AAA progression and reduce vascular calcification. Recently, MM-coated, ROS-responsive NPs (CROR NPs) were engineered to integrate active targeting with environment-responsive drug release [349]. Under high-ROS conditions, CROR NPs undergo structural destabilization, enabling spatiotemporal rapamycin release. This approach effectively mitigated Ca/Pi-induced VSMC calcification, suppressed H_2_O_2_-induced intracellular ROS production and apoptosis, and conferred robust protection against AAA development.

In addition, therapeutic RNA interference strategies have been explored to target matrix degradation. Silencing of MMP-2 and MMP-9 via siRNA delivery has shown potential in inhibiting ECM degradation, stabilizing aneurysmal lesions, and preventing further expansion [350]. These findings underscore the importance of multi-modal nanotherapeutic approaches that combine suppression of inflammation, inhibition of NETs, protection against VSMC apoptosis and calcification, and stabilization of the ECM.

### Treatment of myocarditis

Myocarditis is a pathological condition defined by inflammatory cell infiltration into the myocardium and non-ischemic necrosis of CMs [351]. Unlike systemic vasculopathies that are predominantly associated with AS, myocarditis is most frequently triggered by viral infections, particularly Coxsackievirus B3 (CVB3). Viral infection induces the expression of abnormal cardiac antigens, which are subsequently recognized by T and B lymphocytes, culminating in immune-mediated CMs necrosis [352]. Injury-associated DAMPs released from necrotic CMs bind to pattern recognition receptors (PRRs) on monocytes, stimulating the secretion of chemokines such as CCL2 and macrophage migration inhibitory factor (MIF-α). These mediators orchestrate monocyte recruitment and macrophage activation, amplifying the inflammatory response and driving maladaptive cardiac remodeling [353–356]. Thus, anti-inflammatory therapy constitutes the cornerstone of myocarditis management.

One critical therapeutic target is CCR2, a CCR that mediates the egress of inflammatory monocytes from the bone marrow and directs their recruitment to sites of myocardial injury [357, 358]. Leuschner et al. [359] employed siCCR2-loaded NPs to silence CCR2 expression in bone marrow monocytes, thereby inhibiting the MCP-1/CCR2 axis. This intervention reduced monocyte accumulation at inflammatory sites and indirectly altered the local T-cell response, as diminished monocyte-derived APC activity led to decreased CD4^+^ T-cell infiltration within lesions, collectively attenuating disease progression. Beyond blocking monocyte recruitment, inhibiting their differentiation into pro-inflammatory macrophages further enhances therapeutic efficacy. Monocyte development is dependent on colony-stimulating factor-1 (CSF-1) and its receptor CSF-1R (CD115) [360]. Delivery of siCSF-1 NPs effectively downregulated CSF-1 production in monocytes, disrupting the CSF-1/CSF-1R signaling axis. This led to a substantial reduction in inflammatory monocytes, macrophages, and DCs in the lesion area. The consequent decrease in CD4^+^ T-cell infiltration further alleviated myocardial inflammation, underscoring the potential of targeting monocyte-to-macrophage differentiation pathways in promoting resolution of myocarditis [361]. Future myocarditis therapy should continue to focus on precision anti-inflammatory modulation of immune cells within the CVDs, employing spatiotemporally controlled nanodrug delivery to the bone marrow and myocardium to minimize systemic toxicity. Integrating treatment with immune cell-targeted imaging will enable response-guided dose adjustment and establish a closed therapeutic-diagnostic (theranostic) loop.

## Progress of nanomaterials in cardiovascular clinical translation

Over the past 2 decades, advances in our understanding of the pathophysiological roles of immune cells in CVDs, together with rapid progress in nanotechnology and biotechnology, have substantially accelerated the clinical translation of nanodiagnostic and nanotherapeutic strategies for CVDs [362]. The unique physicochemical properties of nanomaterials, such as their tunable size, surface chemistry, and multifunctionality, have positioned nanomedicine at the forefront of research in disease diagnosis, targeted drug delivery, and molecular therapeutics. Indeed, an increasing number of nanomaterial-based interventions for CVDs have entered clinical trials (Table 3) [363–380].

**Table 3:** Nanomaterials currently applied in clinical research in CVDs

Disease	Nanomaterials	Design	Number of patients	Objectives/Results	Status	Trial numbers	Reference
AS	Silica-gold NPs	Multicenter, randomized, double-blind, interventional	180	Silicon-gold NPs significantly reduced atherosclerotic plaques after being treated with plasma photothermal therapy	Completed	NCT01270139	[363]
	MTX-LDE	Phase II/III, randomized, double-blind, placebo-controlled	40	The LDL-like NPs carrying the anti-inflammatory agent methotrexate have demonstrated safety and efficacy in reducing plaque volume and undesirable characteristics	Unknown status	NCT04616872	[364]
	Paclitaxel -LDE	Phase II/III, prospective, randomized, double-blind, placebo-controlled	40	To evaluate the safety and efficacy of the paclitaxel-LDE for patients with stable coronary artery disease	Unknown status	NCT04148833	[365]
	Silica-gold NP	Phase I, randomized, double-blind, interventional	62	Evaluate the safety and feasibility of the NP delivery technology using microinjection catheters, as well as the efficacy of plasma photothermal therapy combined with stent implantation for AS	Completed	NCT01436123	[366]
	Liposomal prednisolone	Phase I/II, single-center, randomized, placebo-controlled	30	Evaluate the intravenous injection of lipid-based glucocorticoids in patients with an increased risk of AS, and assess the ability to reduce inflammation in the vascular wall	Unknown status	NCT01601106	[367]
	MDCO-216 HDL-like NPs	Phase I/II, proof-of-concept, placebo-controlled, double-blind, randomized	126	MDCO-216 does not cause plaque regression in statin-treated patients	Completed	NCT02678923	[368]
	Polysaccharide SPIO	Phase I, single-center, prospective, interventional	30	The safety of polysaccharide superparamagnetic iron oxide NPs for coronary artery MRI angiography and their efficacy in evaluating the degree of coronary artery stenosis and the stability of the AS plaque	Completed	NCT05032937	[369]
	^18^F-FDG, ^18^F-NaF, USPIO	Observational, prospective, single-arm, prospective	26	Evaluation of the feasibility and value of using PET/CT and USPIO-enhanced MRI for the assessment of AS	Completed	NCT01674257	[370]
	NK-104-NP	Phase I/II, multi-center, open-label, placebo-controlled, interventional	16	NK-104-NP significantly promoted the formation of new blood vessels at the lesion site of the patients, and exhibited a dose-dependent therapeutic effect	Completed	UMIN000008011	[371]
MI	USPIO	Phase II, interventional, single-center	30	USPIO significantly enhances the MRI signal intensity in the MI area, and the R2* values in the infarct and peri-infarct regions increase by 2–3 times	Completed	NCT01995799	[372]
	USPIO	Interventional, single-center	15	Iron NPs can improve MRI signal intensity in the MI region	Completed	NCT01323296	[373]
	^68^ Ga-PRGD2	Phase I, open-label, interventional, single-arm	50	^68^ Ga-PRGD2 can effectively display the distribution and density of αvβ3 integrin in the plaques of patients with MI, and is consistent with the results of MRI imaging	Unknown status	NCT01542073	[374]
IS	Iron NP	Single-center, interventional, open-label	30	The iron NPs are combined with an external rotating magnet, which attracts the NPs into the blood clot, thereby overcoming the effect of blood flow stagnation	Not yet recruiting	NCT06495671	[375]
	USPIO	Phase I, single-arm, open-label, prospective, interventional	5	To evaluate the ability of MRI to track iron oxide-labeled mesenchymal stromal cells after myocardial injection therapy	Completed	NCT03651791	[376]
	^68^ Ga-PRGD2	Phase I, open-label, interventional, single-arm	50	^68^ Ga-PRGD2 can effectively show the vascularization status of patients after IS, and is consistent with the MRI imaging results	Unknown status	NCT01656785	[377]
Acute coronary syndrome	CSL112-HDL	Phase III, multicenter, double-blind, randomized, placebo-controlled, parallel-group	18,226	Compared with the placebo, CSL112 demonstrated a significant difference in reducing the risk of major adverse cardiovascular events within 30 days for patients with acute coronary syndrome	Completed	NCT03473223	[378]
	CER-001 HDL-like NPs	Phase II, multi-center, double-blind, placebo-controlled, dose-focusing	301	No regression of coronary AS	Completed	NCT02484378	[379]
Vascular restenosis	Lip-PTX	Phase II, interventional, randomized, double-blind, multicenter, placebo-controlled	6	Investigation on the preventive effect of Lip-PTX on restenosis after revascularization of the superficial femoral artery	Completed	NCT00518284	[380]

*AS* atherosclerosis, *MTX* methotrexate, *NP* nanoparticles, *MTX-LDE* methotrexate in a cholesterol-rich non-protein NP, *LDL* low-density lipoprotein, *Paclitaxel-LDE* paclitaxel in cholesterol-rich non-protein NPs, *MDCO-216* recombinant apo A-I Milano variant, *SPIO* superparamagnetic iron oxide, *MRI* magnetic resonance imaging, *FDG* fludeoxyglucose, *USPIO* ultrasmall superparamagnetic iron oxide, *PET* positron emission tomography, *CT* computed tomography, *NK-104-NP* pitavastatin-incorporated poly (lactic-co-glycolic acid) NPs, *PEG* polyethylene glycol, ^*68*^* Ga-PRGD2* ^68^ Ga-S-2-(isothiocyanatobenzyl)-1,4,7-triazacyclononane-1,4,7-triacetic acid-PEG3-E[c(RGDyK)]2, *CSL112* apolipoprotein A-I, *HDL* high-density lipoprotein, *CER-001* engineered pre-beta high-density lipoprotein particle, *Lip* liposome, *PTX* paclitaxel

Nevertheless, despite these advances, clinical research on nanomedicines for CVDs remains in its infancy. Most candidates are still in phase I or phase II clinical trials, highlighting the challenges of translating pre-clinical success into clinical efficacy. Unlike conventional drugs, nanomaterials may display unpredictable interactions with biological systems due to their structural and physicochemical complexity, resulting in uncertain pharmacokinetics, biodistribution, and long-term safety profiles [381].

Therefore, the successful clinical translation of nanomaterials requires addressing several critical prerequisites. First, their comparative advantages over standard therapies, in terms of efficacy, specificity, and functional versatility, should be clearly demonstrated. Second, rigorous and systematic evaluation of biocompatibility, pharmacodynamics, and efficacy in relevant animal models is essential. Finally, the feasibility of large-scale manufacturing and quality control should be ensured to support consistent and reproducible clinical application.

### Comparative advantages of diverse nanomaterials in pre-clinical translation

Before discussing the clinical translation prospects of nanomaterials in the diagnosis and treatment of CVDs, it is essential to elaborate on the types, properties, and advantages of nanomaterials currently under investigation in both clinical trials and pre-clinical studies. A comprehensive understanding of CVD pathology, coupled with the unique physicochemical properties of nanomaterials, is critical for designing systems that meet the stringent requirements for clinical translation. Broadly, nanomaterials employed in CVD research can be categorized into polymeric nanomaterials, inorganic nanomaterials, LNPs, and biomimetic nanomaterials [382–385].

#### Polymeric nanomaterials

In CVD diagnosis and therapy, polymeric nanomaterials are primarily engineered as nanospheres, micelles, and nanogels. Their adjustable surface chemistry, structural stability, resistance to systemic degradation, extended circulation half-life, and controlled drug release make them highly attractive for cardiovascular applications [182, 195]. The ease of surface functionalization provides excellent targeting specificity and environmental responsiveness. For example, tailoring polymer type, composition, and particle size allows these nanomaterials to selectively accumulate at diseased sites and respond to abnormal microenvironmental cues [386–389]. Surface modifications, such as PEGylation, enhance immune evasion and prolong systemic circulation [390]. Importantly, their high drug-loading capacity enables multifunctional theranostics, as they can carry both therapeutic molecules and imaging probes simultaneously [184].

A notable subclass of polymers is the dendrimers, which exhibit high solubility, stability, non-immunogenicity, and unique capacity for nucleic acid delivery [391–393]. By forming dendriplexes through electrostatic interactions, dendrimers protect DNA/RNA and efficiently deliver genetic payloads to lesion sites [394]. These platforms have shown promise in modulating immune cell function, inhibiting CVD progression, and promoting local tissue repair and regeneration [194]. Structurally, dendrimers possess internal cavities, low polydispersity, and abundant functional groups, conferring superior drug-loading efficiency compared to conventional polymers [395, 396]. Furthermore, dendrimers can act as templates or stabilizers for embedding imaging agents (e.g., Gd, Ag, Au), thereby enhancing CT or MRI capabilities. Collectively, these features highlight the broad potential of dendrimers as dual diagnostic and therapeutic platforms for CVDs.

#### Inorganic nanomaterials

Inorganic nanomaterials for CVDs can be broadly categorized into metal-based and non-metal-based systems (e.g., mesoporous silica NPs, MSNs; CDs). Metal-based NPs (e.g., Au, Ag, iron oxide) exhibit distinctive physicochemical properties suitable for both imaging and therapy. For instance, superparamagnetic iron oxide NPs (SPIONs) enhance MRI contrast by shortening T_2_ relaxation time, delineating diseased regions as hypointense signals [397]. Gold NPs serve as efficient CT contrast agents for vascular stenosis and aneurysms [398], and they also exhibit antioxidant and anti-hypertrophic properties, suggesting cardioprotective applications [399]. Their modifiable surfaces allow functionalization with targeting ligands to direct interactions with immune cells [400]. Non-metal-based nanomaterials, including carbon- and silicon-based NPs, leverage their large surface area, tunable particle size, and functionalizable surfaces to achieve high drug-loading capacity and superior biocompatibility [401]. CDs, in particular, possess unique optical properties that support photoacoustic and fluorescence imaging, offering multifunctional theranostic opportunities in CVDs [402].

#### LNPs

LNPs are among the most clinically advanced nanocarriers, owing to their biocompatibility, biodegradability, and low immunogenicity [403]. They efficiently encapsulate both hydrophobic and hydrophilic drugs, enhancing solubility, dispersibility, and controlled release. Moreover, their surfaces can be functionalized for active targeting and stimuli-responsive delivery, enabling focused therapy for CVDs such as MI, coronary thrombosis, and AS [404, 405]. Through ligand modification and PEGylation, LNPs have evolved from traditional long-circulating systems to smart carriers capable of dynamic responses to pathological microenvironments [406, 407]. In recent years, mRNA-based therapeutics have shown great potential in modulating angiogenesis, CM proliferation, fibrosis reduction, and tissue regeneration [408, 409]. LNPs represent the clinically approved platform for mRNA delivery, protecting nucleic acids from enzymatic degradation and mitigating the immunogenicity associated with naked mRNA, while enhancing delivery efficiency in cardiovascular settings [410, 411].

#### Biomimetic nanomaterials

Biomimetic nanomaterials used in CVDs are essentially nanomaterials functionalized with cell membranes. These membranes can be derived from various cell types, including red blood cells, macrophages, neutrophils, T cells, or secreted exosomes [190, 197, 227]. This strategy is often referred to as the “Trojan horse” approach. By being cloaked in a homologous cell membrane, these NPs gain a “self-recognition” ability, which significantly reduces their chances of being identified and cleared by the immune system. This, in turn, markedly prolongs their circulation time in the bloodstream [412]. The cell membrane also provides homologous targeting capabilities, enabling the nanomaterials to actively target specific sites. For example, NPs that mimic macrophages can specifically bind to sites of vascular injury and ruptured atherosclerotic plaques, as these areas expose specific adhesion molecules [181]. This active targeting capability is far more potent than the passive EPR effect. Furthermore, the cell membrane camouflage allows these nanomaterials to cross the BBB, precisely targeting areas in the brain [413–416]. This promotes drug accumulation within cerebral lesions, giving them immense potential for the diagnosis and treatment of IS [417].

### Clinical safety issues

The foremost challenge in the clinical translation of nanomedicines for CVDs lies in biosafety [418]. Although candidate nanomaterials are generally selected for their inherent biocompatibility, their nanoscale dimensions and intimate interactions with the bloodstream and vascular endothelium introduce unique cardiovascular risks [419]. For example, while gold is traditionally considered “bioinert”, gold NPs have been reported to induce DNA damage under certain conditions [420]. Similarly, although carbon NPs (CNPs) and carbon nanotubes (CNTs) are widely utilized in other biomedical fields, pharyngeal aspiration of single- or double-walled CNTs in murine models significantly increased monocyte adhesion to ECs, accelerating atherogenesis [421]. Silica-based nanomaterials have been shown to trigger pyroptosis and promote cardiac hypertrophy [422]. Likewise, iron-based NPs, such as ultrasmall SPIOsNPs (USPIOs), commonly applied for imaging myocardial and vascular inflammation, have been associated with thrombotic reactions in vivo, platelet aggregation in vitro, DNA damage, and heightened oxidative stress in CMs, thereby exerting adverse effects that contrast with the relative safety of larger Fe_3_O_4_ NPs [423–426]. Furthermore, despite their functional advantages, cell-membrane-camouflaged biomimetic NPs may elicit immunogenic responses, raising additional translational concerns [238].

Given these risks, a comprehensive biological evaluation is indispensable before clinical application. Such assessments must include detailed analyses of biodistribution, metabolism, immunological effects, and toxicological outcomes. NP toxicity remains a multiscale challenge, spanning from organ-level pathology to cellular and even subcellular perturbations within the nucleus. To address this, biocompatibility must remain a primary design criterion, complemented by extensive in vitro toxicological testing. Optimizing particle size, surface charge, morphology, and administration route can substantially mitigate cytotoxicity [174, 427–430]. Following in vivo application, a complete biosafety profile should be established through hematological analyses, histopathological examination of major organs, and evaluation of immune activation and immunotoxicity.

A major contributor to nanotoxicity is the off-target effect on non-diseased tissues and cells. Thus, enhancing the targeting specificity of nanomaterials represents a critical direction for future research to minimize systemic toxicity. Moreover, given the chronic nature of CVDs, more convenient and patient-compliant administration routes, such as oral or transdermal delivery systems, should be explored to overcome the limitations of current intravenous administration strategies [383, 431–433].

### Validity of animal models

Given the biosafety concerns associated with nanomaterials, their translational efficacy from animal models to humans requires careful evaluation. Although nanomaterials have demonstrated excellent diagnostic and therapeutic efficacy in controlled laboratory settings, successful pre-clinical outcomes often fail to replicate in human clinical trials [434]. A primary factor underlying this translational gap is the substantial biological divergence between animal models and human CVDs.

First, most experimental models are established over relatively short durations (typically several weeks), which does not faithfully recapitulate the chronic, heterogeneous, and multifactorial progression of human CVDs [435–438]. Second, human cardiovascular pathologies are strongly influenced by diverse genetic backgrounds, aging, sex differences, environmental exposures, and comorbidities such as hypertension, diabetes, systemic inflammation, and obesity [439–442]. For instance, Apoe^−/−^ mice spontaneously develop AS due to impaired clearance of apoE-containing lipoproteins, regardless of cholesterol feeding, whereas human AS is usually multifactorial and heavily environment-dependent [440]. Similarly, in humanized human cholesterol ester transfer protein (hCETP) transgenic mice with di- and tri-genetic hypertriglyceridemia [Tg (hApoC3 × hCETP) and Tg (hApoA1 × hApoC3 × hCETP)], early atherosclerotic lesion formation was unexpectedly inhibited, contradicting epidemiological evidence in humans [443]. Conversely, introduction of the same genetic modification into a Dahl salt-sensitive hypertensive rat to generate a transgenic polygenic hypertension model [Tg (hCETP)^DS^] revealed that hypertriglyceridemia exhibited a pro-atherosclerotic effect consistent with human epidemiology [444]. These findings highlight how even identical genetic alterations may produce divergent disease phenotypes across species, thereby questioning the translational reliability of current animal models.

Future nanomaterial research for CVDs must therefore prioritize the refinement of pre-clinical models. One strategy involves the use of humanized animal models, such as the APOE*3-Leiden. CETP mouse, which more accurately recapitulates human lipoprotein metabolism and thus provides a more reliable platform for simulating human AS and related pathologies [445]. Moreover, implementing moderately prolonged cholesterol feeding regimens may better reflect chronic hyperlipidemia and the long-term disease courses observed in patients [436, 446–448]. In parallel, deeper investigation into genetic alterations underlying human CVDs will facilitate the identification of clinically relevant therapeutic targets and the design of nanomaterials tailored to human pathophysiology.

Given the variability among animal models, it is essential to adopt a multi-model approach, integrating findings across species to strengthen translational validity. Beyond traditional models, advanced technologies such as organ-on-a-chip systems and organoids offer innovative platforms that more faithfully simulate human tissue microenvironments, cellular heterogeneity, and dynamic physiological processes [449–451]. These emerging tools hold great potential to bridge the translational gap, accelerating the pathway from bench to bedside in the clinical development of cardiovascular nanomedicines.

### Complexity in nanomaterials construction

The manufacturing complexity of nanomaterials remains a critical barrier to their clinical translation. Despite promising outcomes in laboratory research, the majority of nanomaterials developed for the diagnosis and treatment of chronic conditions such as CVDs remain at the pre-clinical stage. Their production typically depends on time-intensive, technically demanding, and low-yield processes, which are often associated with poor quality control and significant batch-to-batch variability in key parameters, including particle size, drug-loading capacity, structural stability, and overall yield [174]. Biomimetic nanomaterials represent a particular challenge. Their fabrication requires the integration of cell membranes or other biological components, yet the absence of standardized manufacturing protocols results in inconsistent reproducibility. In addition, these systems frequently display limited stability and inadequate long-term storage properties, further restricting their translational potential [452]. Similarly, dendrimers, although structurally versatile and therapeutically promising, suffer from intricate and multistep synthesis procedures, which hinder scalability and limit the feasibility of industrial-scale production [453].

To overcome these challenges, once nanomaterials demonstrate acceptable biocompatibility, therapeutic efficacy, and reliable translational performance from animal to human models, the development of scalable and reproducible manufacturing processes becomes the decisive step toward clinical applications. Future nanomaterial design should therefore prioritize simplicity, robustness, and translational feasibility. Streamlining formulations and minimizing unnecessary structural complexity not only facilitates manufacturing but also reduces the risk of toxicity associated with overly complex architectures. Moreover, advances in automated synthesis and microfluidics offer promising avenues to address current limitations. These technologies enable precise control over reaction parameters, resulting in the rapid, efficient, and reproducible fabrication of NPs with uniform size, shape, and drug-loading capacity [454]. Integrating such approaches into the production pipeline may significantly enhance yield, consistency, and scalability, thereby advancing the clinical readiness of nanomaterial-based therapies for CVDs.

## Conclusion and perspective

Nanotheranostics, which integrates diagnostic and therapeutic functionalities at the nanoscale, represents a transformative paradigm for CVD management. By selectively modulating immune cell responses, particularly those of macrophages and neutrophils, nanomaterials offer novel strategies to suppress pathological inflammation while simultaneously promoting tissue repair in conditions such as AS, MI, and aneurysms. Meanwhile, immune cell-targeted imaging platforms enable real-time visualization of lesion biology and treatment response, allowing dynamic tracking of biomarkers before and after interventions. This dual diagnostic-therapeutic model not only enhances precision in treatment planning but also strengthens the foundation for personalized cardiovascular care [455]. Despite these advances, several critical challenges remain in nanomedicine-based cardiovascular immunotherapy: (1) Limited immune cell coverage: most nanomaterial designs to date have focused primarily on macrophages and neutrophils, while other key immune regulators, such as mast cells, DCs, T cells, and B cells, have received relatively little attention. Given that CVD pathology arises from the synergistic dysregulation of multiple immune subsets, single-target nanotherapeutics are unlikely to effectively resolve the complex inflammatory milieu [12, 456]. Future efforts should thus prioritize multi-targeted nanomaterials capable of simultaneously regulating multiple immune cell populations and reprogramming the aberrant inflammatory microenvironment. Such a multi-pronged immunomodulatory approach will more effectively suppress the core drivers of chronic inflammation and facilitate long-term tissue repair. (2) Integration of imaging and therapy: coupling ultrasensitive imaging modalities with therapeutic nanoplatforms provides unique opportunities for real-time monitoring of disease progression and treatment efficacy. For example, Wang et al. [457] developed metal-free nanozymes (HCN@DS) that suppress AS progression while enabling photoacoustic/photothermal imaging-guided macrophage autophagy activation. Compared to conventional regimens requiring separate imaging agents, theranostic platforms reduce redundancy, improve accuracy, and enable dynamic treatment optimization. Future designs should emphasize lightweight, multifunctional nanomaterials that seamlessly combine diagnostic precision with therapeutic potency. (3) Emerging technologies to enhance translation: although nanomaterial-based immunotherapies have achieved remarkable pre-clinical success, their clinical translation remains limited. Emerging technologies provide powerful tools to overcome current bottlenecks. CRISPR-based immunomodulation, particularly when delivered by LNPs, enables gene-level editing of immune responses, offering unprecedented precision in dampening lesion-specific inflammation while mitigating issues of instability, poor uptake, and immunogenicity [382, 458]. In parallel, the incorporation of deep learning algorithms into cardiovascular imaging can enhance signal-to-noise ratio, improve spatial resolution, and facilitate quantitative and predictive analysis of therapeutic outcomes [459]. Future nanomaterials should therefore be conceived as integrated platforms, uniting immune regulation, genetic modulation, and AI-assisted imaging to achieve patient-specific nanomedicine guided by individualized immunological and genetic profiles. (4) Balancing efficacy and safety: a major limitation of current nanotherapeutics lies in the potential for systemic immunosuppression, which may impair host defense or delay repair processes [460]. To overcome this, next-generation designs should emphasize selective immune modulation rather than broad suppression. Localized and spatiotemporally controlled delivery systems that combine anti-inflammatory and pro-regenerative cues will be crucial to achieving both therapeutic precision and biosafety.

In summary, nanomaterial-mediated immunomodulation demonstrates tremendous potential in reshaping the landscape of CVD therapy. The minimally invasive nature, multifunctionality, and targeting precision of nanotechnology confer unique advantages for addressing the complex and heterogeneous pathophysiology of CVDs. By acting on lesion-resident immune cells, nanomaterials achieve etiological treatment through 3 interconnected mechanisms: 1) functional reprogramming of immune cells, 2) neutralization of pro-inflammatory mediators, and 3) remodeling of local inflammatory niches. Together, these actions halt disease progression at its roots, aligning with the systemic treatment philosophy required for panvascular disorders [461]. Future research, building on a deepened understanding of immune cell dynamics in cardiovascular pathologies, will likely expand nanotheranostic applications across the disease spectrum. The integration of prevention, diagnosis, treatment, and management into unified nanoplatforms promises to realize precision medicine throughout the cardiovascular care continuum.

## Data Availability

No applicable.

## Abbreviations

CVDs: Cardiovascular diseases
AS: Atherosclerosis
MI: Myocardial infarction
IS: Ischemic stroke
ASCVDs: Atherosclerotic cardiovascular diseases
IL: Interleukin
TNF: Tumor necrosis factor
NF-κB: Nuclear factor kappa-B
EC: Endothelial cell
SMCs: Smooth muscle cells
ECM: Extracellular matrix
CCR: Chemokine receptor
ICAM: Intercellular cell adhesion molecule
DC: Dendritic cell
NLRP3: NOD-like receptor thermal protein domain associated protein 3
MMPs: Matrix metalloproteinases
ROS: Reactive oxygen species
NO: Nitric oxide
FCs: Foam cells
ABCG1: ATP binding cassette subfamily G member 1
SR: Scavenger receptor
TGF: Tumor growth factor
CXCR: C-X-C chemokine receptor type
LDL: Low-density lipoprotein
MPO: Myeloperoxidase
NETs: Neutrophil extracellular traps
VSMCs: Vascular smooth muscle cells
NE: Neutrophil elastase
Th: T helper
FPR2: N-formyl peptide receptor 2
EGF: Epidermal growth factor
VEGF: Vascular endothelial growth factor
Tregs: Regulatory T cells
FOXP3: Forkhead box protein P3
MCP-1: Monocyte chemoattractant protein-1
APCs: Antigen-presenting cells
MCs: Mast cells
HDL: High-density lipoprotein
NK: Natural killer
ox-LDL: Oxidized LDL
siRNA: Small interfering RNA
MM: Macrophage membrane
RES: Reticuloendothelial system
IRI: Ischemia-reperfusion injury
IRF1: Interferon regulatory factor 1
FLI: Fluorescence imaging
PAI: Photoacoustic imaging
DBCO: Azide-dibenzocyclooctyne
CDs: Carbon dots
BBB: Blood-brain barrier
PLM: Platelet cell membranes
AAA: Abdominal aortic aneurysm
NP: Nanoparticle
PEG: Polyethylene glycol
PDA: Polydopamine
CMs: Cardiomyocytes
LXR: Liver X receptor
VCAM: Vascular cell adhesion molecule
PET: Positron emission tomography
MRI: Magnetic resonance imaging
M1: Classically activated macrophage phenotype
M2: Alternatively activated macrophage phenotype
CT: Computed tomography
CXCL: C-X-C motif chemokine ligand
IFN: Interferon
CCL: C-C motif chemokine ligand
VEGFA: Vascular endothelial growth factor A
FPR2/ALX: Formyl peptide receptor 2/ lipoxin A_4_ receptor
EMMPRIN: Extracellular matrix metalloproteinase inducer
SPRY1: Sprouty receptor tyrosine kinase signaling antagonist 1
LaCD: Luminol-conjugated α-cyclodextrin material
hCETP: Human cholesterol ester transfer protein
MerTK: MER tyrosine kinase
